# The Cologne Picture Naming Test for Language Mapping and Monitoring (CoNaT): An Open Set of 100 Black and White Object Drawings

**DOI:** 10.3389/fneur.2021.633068

**Published:** 2021-03-03

**Authors:** Carolin Weiss Lucas, Julia Pieczewski, Sophia Kochs, Charlotte Nettekoven, Christian Grefkes, Roland Goldbrunner, Kristina Jonas

**Affiliations:** ^1^Center for Neurosurgery, Faculty of Medicine and University Hospital, University of Cologne, Cologne, Germany; ^2^Department of Neurology, Faculty of Medicine and University Hospital, University of Cologne, Cologne, Germany; ^3^Department of Special Education and Rehabilitation, Faculty of Human Sciences, University of Cologne, Cologne, Germany

**Keywords:** picture naming, neuromonitoring, intraoperative, TMS, language, brain tumour, assessment, German

## Abstract

Language assessment using a picture naming task crucially relies on the interpretation of the given verbal response by the rater. To avoid misinterpretations, a language-specific and linguistically controlled set of unambiguous, clearly identifiable and common object–word pairs is mandatory. We, here, set out to provide an open-source set of black and white object drawings, particularly suited for language mapping and monitoring, e.g., during awake brain tumour surgery or transcranial magnetic stimulation, in German language. A refined set of 100 black and white drawings was tested in two consecutive runs of randomised picture order and was analysed in respect of correct, prompt, and reliable object recognition and naming in a series of 132 healthy subjects between 18 and 84 years (median 25 years, 64% females) and a clinical pilot cohort of 10 brain tumour patients (median age 47 years, 80% males). The influence of important word- and subject-related factors on task performance and reliability was investigated. Overall, across both healthy subjects and patients, excellent correct object naming rates (97 vs. 96%) as well as high reliability coefficients (Goodman–Kruskal's gamma = 0.95 vs. 0.86) were found. However, the analysis of variance revealed a significant, overall negative effect of low word frequency (*p* < 0.05) and high age (*p* < 0.0001) on task performance whereas the effect of a low educational level was only evident for the subgroup of 72 or more years of age (*p* < 0.05). Moreover, a small learning effect was observed across the two runs of the test (*p* < 0.001). In summary, this study provides an overall robust and reliable picture naming tool, optimised for the clinical use to map and monitor language functions in patients. However, individual familiarisation before the clinical use remains advisable, especially for subjects that are comparatively prone to spontaneous picture naming errors such as older subjects of low educational level and patients with clinically apparent word finding difficulties.

## Introduction

The correct identification and semantic retrieval of object names in a behavioural task is the basis of investigating conceptual knowledge of objects in the human brain (1). When using an overt object naming task, also expressive speech motor functions (i.e., articulation) are involved. This task, therefore, combines important language domains, which might have led to its wide use in the assessment and monitoring of language functions, e.g., for language mapping and monitoring in the context of awake neurosurgery (2). Controlling the correctness of the verbal answer is essential to assess either object identification, lexical/semantic retrieval, or word articulation. Different linguistic factors are known that affect the ease of the retrieval process and task performance in general. Three of these important factors are addressed in this work:

First, the uniqueness of the object drawing to be named and the disambiguity of the corresponding word to be retrieved are crucial pre-requisites of reliable testing and calls for objects that can be easily depicted graphically as well as for the non-existence of alternative expressions (i.e., synonyms) to name the respective object [see (3) for review]. Second, word frequency, i.e., how often a certain word is typically used in a certain language, is described as an objective and highly relevant factor influencing lexical access in naming tasks [e.g., (4) for review, (5, 6)], given the association of higher frequency words with a lower error rate as well as with faster retrieval process (6). A third relevant factor is the word length, here expressed by the number of syllables, since longer words are associated with a higher error rate (7). All factors vary, however, with respect to age or educational level as well as cultural background and language so that existing stimuli and procedures cannot be directly transferred from one language to another (8, 9).

Although overt object naming tasks are widely used in both neurocognitive science and clinical practise, linguistically controlled and validated open-source assessment tools are scarce. As a result, to date, there is no consensus tool for intraoperative monitoring of language functions during awake surgery of cerebral lesions or related pre-surgical investigations, especially for the German language. Providing a linguistically controlled and validated stimulus set for use in German language might be of great value, e.g., to allow for data comparison in multicentre studies and to assure a state-of-the-art testing procedure, robust to possibly erroneous interpretations due to low reliability of the test protocol itself.

In the context of neurosurgery, the precise delineation of the boundaries of eloquent brain areas by intraoperative direct cortical stimulation (DCS) is extremely important not only to achieve maximum tumour control and improve survival but also to avoid permanent neurological deficits (10). For language, this is particularly relevant since the anatomical correlates of function underlie a much higher variability as compared to, e.g., primary motor functions, in both healthy (11) and, even more, in diseased brain (12–15).

Since its introduction by Penfield and Roberts (16), visual object naming has become the most common task for intraoperative language mapping and monitoring (17). Apart from its inclusion in neuropsychological and language-related assessment batteries and its use for non-invasive functional imaging [e.g., magnetoencephalography, functional magnetic resonance imaging and positron emission tomography; (18–20)], the object naming task has also been used for neuronavigated, repetitive, task-locked transcranial magnetic stimulation (TMS). This technique simulates the intraoperative situation during awake surgery where task execution is temporarily hampered by local electrical stimulation (i.e., DCS) of a cortex site, also referred to as “virtual lesion” (21–23).

Like neurocognitive and language assessment for diagnostic purposes, the results of both TMS and DCS rely crucially on the *ad hoc* (intraoperative) or *post-hoc* (post-operative) interpretation of the given verbal response by the rater. Here, a language-specific and linguistically controlled set of unambiguous, clearly identifiable and common object–word pairs is particularly important.

Existing stimulus sets are of limited usability for German-speaking subjects due to language specificity of the normative data and/or the stimuli, mostly designed for English native speakers [e.g., (24–26)], and/or due to copyright protection [e.g., (27, 28)]. We, therefore, set out to validate and provide an open-source set of black and white object drawings, specifically for German-speaking subjects, intended for both research and clinical use: The Cologne Picture Naming Test for Language Mapping and Monitoring (CoNaT). We expected high correct object naming rates and a strong correlation between the given answers and hypothesised that both word-related linguistic characteristics, i.e., higher number of syllables and lower word frequency, have a significant negative impact on object naming performance. Moreover, we expected better task performance from subjects of young age and high educational level. Apart from investigating the robustness of the task and the influence of these word- and subject-related factors on the object naming performance in a representative cohort of healthy adults of all age groups, we also assessed the suitability of the CoNaT as a reliable language monitoring instrument in a pilot cohort of brain tumour patients.

## Materials and Methods

### General Study Design

A set of 112 black and white drawings was tested in respect of correct object identification as well as correct, prompt and reliable object naming in a representative series of 132 healthy subjects and a clinical pilot cohort of 10 brain tumour patients.

For the development of the picture set, we generally included concrete monomorphematic simple nouns (no compound nouns) for which a clear and unambiguous pictorial illustration was feasible (29). In addition, two linguistic factors (i.e., word frequency, number of syllables) were considered to build four equally large subgroups of object–word pairs (see Stimuli Set section).

We set out to assess (i) the feasibility as expressed by the overall rate of correctly identified items and (ii) the test–retest reliability of the object naming performance, both of which are important to qualify the CoNaT e.g. for intraoperative monitoring, as well as (iii) the influence of stimulus- and subject-related characteristics on correct object recognition and naming reliability. Moreover, we investigated whether or not a correlation between object naming performance and the test result of a standard assessment of word finding difficulties (i.e., Bielefeld Screening for word finding difficulties for mild aphasia [BIWOS]; (29)) could be found in the pilot cohort of patients with utmost mild to moderate clinical signs of aphasia. Both groups, healthy subjects and brain tumour patients, performed the naming task twice, in two consecutive runs.

The study was carried out according to the declaration of Helsinki [(30), last revision 2013] and was approved by the local ethics committee.

### Subjects

#### Healthy Subjects

A total of 132 healthy subjects between 18 and 84 years of age were prospectively enrolled between 2016 and 2019. Subjects were characterised by age (group 1: 18–35 years; group 2: 36–53 years; group 3: 54–71 years; group 4: 72 years or older), gender, handedness, and general educational level (i.e., holding vs. missing university entrance diploma, generally corresponding to ≥/ <12 years of general school education). Here, technical college entrance qualification was considered as equivalent to a university entrance diploma. Inclusion criteria were as follows: age of at least 18 years; German language skills on native speaker level; no intake of alcohol, drugs or psychoactive agents prior to the experiment with risk of reduced attention and/or alertness levels; and sufficient vision (i.e., ≥0.7 corrected visual acuity). Subjects with neurological or psychiatric diseases (including brain lesions and seizures) in medical history were excluded.

#### Patients

In addition, 10 adult patients with clinical signs of mild to moderate aphasia were included in this study in order to test the protocol under clinical conditions. All patients were newly diagnosed with a focal brain tumour of the left hemisphere.

The additional inclusion criteria were identical for both healthy subjects and patients. In contrast, specific exclusion criteria for patients were as follows: (i) neurological/psychiatric diseases unrelated to the brain tumour, (ii) clinical signs of moderate to severe cognitive dysfunction as indicated by a Mini Mental State Examination [MMSE; (31)] score of <20/30, and (iii) severe word finding difficulties according to a screening of object naming competence using 10 pictures (which were not included in the protocol). Here, correct naming of at least 7 out of the 10 objects was required to qualify for study inclusion.

The severity of word finding difficulties of all participating patients was characterised using the BIWOS assessment. Of note, the BIWOS was chosen since it tests for a comprehensive set of semantic and lexical language skills for diagnosing word finding difficulties by a series of well-standardised tasks (i.e., antonyms, rhymes [free, category specific], hyperonyms, verbal fluency [lexical, semantic], word composition, semantic feature analysis, naming by definition) but does not include visual object naming so that a low level of interference was expected. The BIWOS was analysed according to the standard procedure given in the manual, resulting in separate scores for lexical and semantic word finding skills as well as a total score and corresponding severity levels to describe the word finding difficulties.

Of note, all complementary examinations (i.e., MMSE, screening of object naming competence, BIWOS) were administered prior to the beginning of the object naming tests.

### Stimuli Set

The entire picture set (*N* = 112) consisted of four different categories (A–D as defined by number of syllables and high vs. low word frequency) and included a total of 12 back-up illustrations to allow for a posteriori selection of the 100 best suited pictures (Table 1). All object–word pairs were chosen based on the pilot data by a clinical neuroscientist together with an experienced linguist (i.e., authors CWL and KJ) and were controlled regarding the following criteria: (i) (gender neutral) word frequency [cf. (33, 34)], (ii) number of syllables, and (iii) unambiguity of both the object illustration and the expected verbal response (i.e., good recognizability of the illustrated object, expected non-existence of synonyms for the object name in German language as well as the absence of semantically related attributes, which could lead to compound nouns and over-specified verbal responses such as “egg cup” instead of “egg”).

**Table 1:** Stimulus set characteristics.

**Class**	**Number of syllables**	**Word frequency**		**Number of stimuli**	
		**Category**	**Median [range]**	**Tested**	**Selected**
A	1	High	53 [14–729]	28	25
B	2	High	24 [11–170]	28	25
C	1	Low	5 [1–10]	28	25
D	2	Low	4 [0–9]	28	25
Total	1–2		11 [0–729]	112	100

*Word frequencies are given according to the CELEX database (http://celex.mpi.nl) (32) of word frequencies in German language. Word frequencies > 10/1,000,000 words were defined as high*.

**Figure 1:**
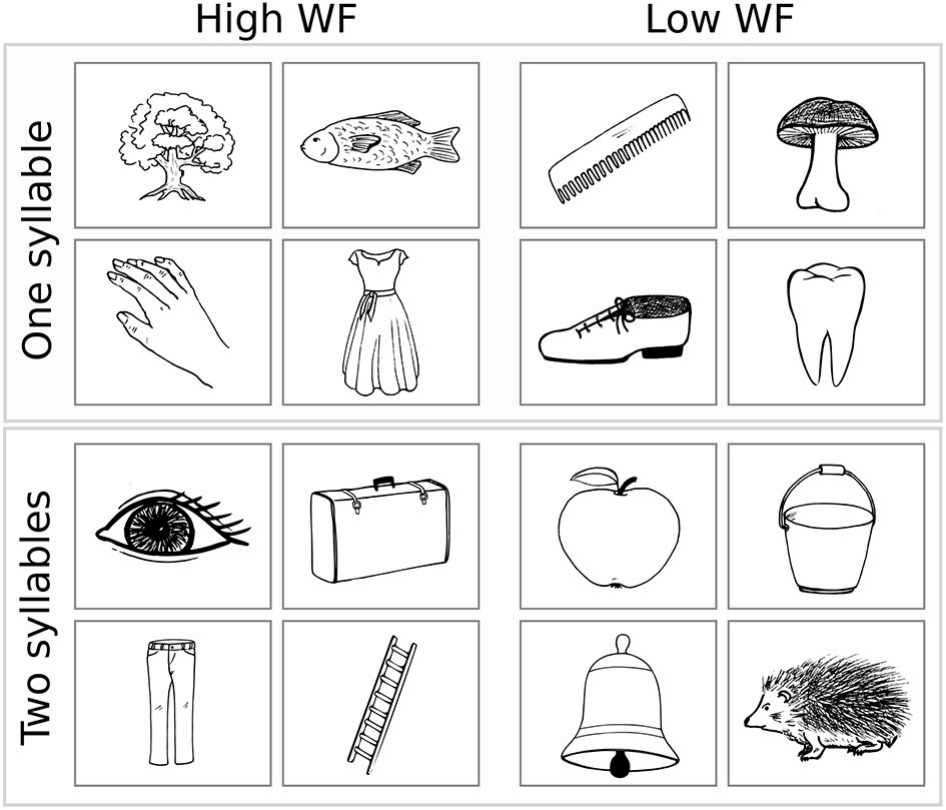
Example stimuli. Four example drawings are given for class A (upper left) to D (lower right). WF, Word frequency.

Illustrations were black and white drawings (presented on a white screen), drawn by author CWL and were either (i) freely designed (*n* = 53) or inspired (ii) by the Snodgrass & Vanderwart picture set [*n* = 25; (24)] or (iii) by the pictures included in the commercial software Nexspeech (Nexstim Oy, Helsinki, Finland; *n* = 22). A total of *n* = 12 drawings (i.e., three drawings per class A–D) were omitted due to poor performance in respect of either correctness or unambiguity of the naming responses (mean correct naming rate: 87 ± 7%; mean Goodman and Kruskal's gamma [referred to as “GK-gamma” throughout the manuscript]: 0.94 ± 0.05) and were, thus, not considered for further statistical analysis (see Supplementary Table 1 for details). The remaining selection of *n* = 100 objects is provided in Table 2 (see Supplementary Material for stimuli, i.e., drawings). Example drawings are shown in Figure 1.

**Table 2:** Word lists.

**A**		**B**		**C**		**D**	
**(One syllable, high WF)**		**(Two syllables, high WF)**		**(One syllable, low WF)**		**(Two syllables, low WF)**	
**Object name**	**WF**	**Object name**	**WF**	**Object name**	**WF**	**Object name**	**WF**
Arm [arm]	57	Auge [eye]	56	Blitz [lightning]	8	Apfel [apple]	6
Bank [bank]	85	Auto [car]	78	Bus [bus]	7	Birne [pear]	0
Baum [tree]	24	Brille [glasses]	17	Ei [egg]	9	Blume [flower]	3
Bett [bed]	80	Engel [angel]	27	Fass [barrel]	3	Bürste [brush]	2
Brot [bread]	28	Feder [feather]	11	Frosch [frog]	1	Drache [dragon]	2
Buch [book]	99	Fenster [window]	75	Kamm [comb]	5	Eimer [bucket]	5
Fisch [fish]	17	Finger [finger]	42	Knopf [button]	6	Gabel [fork]	4
Fuß [foot]	49	Hose [trousers/pants]	11	Kran [crane]	5	Glocke [bell]	0
Glas [glass]	60	Insel [island]	29	Maus [mouse]	5	Harfe [harp]	1
Hand [hand]	316	Kette [chain]	17	Pfeil [arrow]	7	Hase [rabbit]	7
Haus [house]	104	Kirche [church]	170	Pilz [mushroom]	1	Igel [hedgehog]	5
Herz [heart]	79	Koffer [suitcase]	18	Rock [skirt]	10	Käse [cheese]	6
Hund [dog]	35	König [king]	84	Schal [scarf]	1	Katze [cat]	9
Hut [hat]	14	Krone [crown]	18	Schuh [shoe]	5	Kerze [candle]	3
Kleid [dress]	29	Leiter [ladder]	56	Schwamm [sponge]	2	Löffel [spoon]	6
Kuh [cow]	23	Löwe [lion]	11	Schwein [pig]	5	Messer [knife]	7
Mund [mouth]	53	Mauer [wall]	35	Schwert [sword]	7	Muschel [mussel]	1
Pferd [horse]	29	Schlange [snake]	11	Ski [ski]	10	Puppe [doll]	5
Rad [wheel]	25	Schlüssel [key]	23	Storch [stork]	4	Säge [saw]	2
Schloss [padlock]	64	Sonne [sun]	90	Topf [pot]	7	Schaukel [swing]	0
Stern [star]	34	Teppich [carpet]	24	Wurst [sausage]	9	Schere [scissor]	4
Stuhl [chair]	26	Teufel [devil]	24	Zahn [tooth]	2	Schleife [bow]	6
Tisch [table]	89	Trommel [drum]	24	Zaun [fence]	10	Spritze [syringe]	5
Tür [door]	113	Vogel [bird]	24	Zelt [tent]	6	Wecker [alarm clock]	0
Uhr [clock]	729	Zeitung [newspaper]	24	Zwerg [dwarf]	2	Würfel [dice]	3

*Object names are given in German [English translation]. WF, word frequency in German*.

### Test Protocol and Scoring

Pictures were presented in a pseudorandomised sequence on a white screen. The display time for each stimulus was 500 ms, interleaved by a time interval of 3 s for healthy subjects and 5 s for patients. No feedback was provided regarding the task performance (i.e., correctness of picture naming) during the experiment. Between the two consecutive sessions, a break of up to 10 min was allowed if required by the test subject, e.g., in case of tiring.

For each run, the verbal responses were audio-taped for additional *post-hoc* assessment of promptness, accuracy, and reliability of object recognition and naming (Table 3) to account for both the uniqueness of the illustration and the unambiguity/simplicity of the semantic word retrieval and its articulation. Here, more specific object names compared to the expected verbal response like “sparrow” instead of “bird” as well as compound nouns instead of simple nouns such as “church bell” for “bell” were rated as over-specification and thus fell into the category of unexpected naming variants (i.e., category III, cf. Table 3). In contrast, generalisations like “animal” instead of “bird” were categorised as wrong naming response (i.e., category V; cf. Table 3). Response delays were assessed by acoustic evaluation, a common procedure in clinical practise (e.g., for pre-surgical and intraoperative language mapping using TMS/DCS), hereby considering the individual baseline response latency. For further analyses, correct responses were assigned to the types (A) “correct object naming,” including only correctly recognised and expectedly named objects (i.e., categories I–II), and (B) “correct object recognition,” including also correctly recognised but unexpectedly named objects (i.e., categories I–III; Table 3).

**Table 3:** Verbal response rating.

**Verbal response**		**Correct performance type**	
**Category**	**Description**	**“Naming”**	**“Recognition”**
I	Prompt and correct as expected	x	x
II	Correct as expected but response delayed	x	x
III	Unexpected naming variant, e.g., dialect or cultural synonym, over-specification, diminutive or plural		x
IV	Wrong but self-correction		
V	Wrong or non-response		

*Overt naming responses were categorised as follows: (i) prompt and correct, (ii) correct but delayed, (iii) unexpected naming variants like dialectal, cultural, or other previously unexpected synonyms (e.g., “Beelzebub” instead of “devil”), over-specification, diminutive, or plural, (iv) wrong but self-correction, and (v) wrong or non-response, with (i–ii) being considered as correct object recognition expressed by a correct and expected naming response (referred to as “correct naming response”) and (i–iii) being considered as correct object recognition, including both expected and unexpected naming responses (referred to as “correct objection recognition” throughout the manuscript)*.

### Statistics

Normality of data distributions was tested according to Shapiro–Wilk. The reliability of naming performance (categorical data; five levels; see above) between the first and the second run was assessed using GK-gamma for each stimulus item.

An analysis of variance (ANOVA) was performed to test for the influence of stimulus- and subject-related factors on the results, i.e., on the average rate of correct object recognition as well as correct object naming (two levels: right vs. wrong; see above) in percent of total trials and the reliability of the naming responses (five levels i–v; Table 3) as expressed by GK-gamma. GK-gamma is a symmetric measure of association, based on a sorted list of paired observations, which ranges from −1.0 to +1.0, with +1.0 indicating perfect correlation. Please note that, for the ANOVA and for calculation of correlations, GK-gamma = 1 was assumed if GK-gamma could not be calculated due to perfect naming rates (i.e., 100% correct naming in both sessions). For ANOVA with GK-gamma as dependent (outcome) variable, outliers (i.e., >2 SD deviation from average) were omitted. Of note, this outlier removal had to be applied only for subjects/stimuli where the confidence interval was zero due to very low incidence of errors. In total, this procedure removed 10% (subjects)/13% (stimuli) of the total data. Levels of significance according to ANOVA are indicated without leading zeros (e.g., “*p* < 0.01”) throughout the manuscript to allow for better distinction from results of group mean comparisons and correlations.

*Post*-*hoc* comparison of means between paired data (e.g., correct naming rates of session 1 vs. session 2) were calculated using paired *t*-tests or Wicoxon's signed rank test, depending on the normality of the data distribution (as assessed by the Shapiro–Wilk test). Accordingly, for comparison between independent groups, Wicoxon's rank test was applied in case of not normally distributed data.

Pearson's correlation was calculated to test for significant relationships between metric variables (i.e., behavioural scores).

In cases of comparisons between more than two groups (e.g., between different word groups: A–D), the levels of significance were adjusted using the false discovery rate (FDR) correction (35).

The statistical analysis was performed using R (R Studio, Version 0.98.507, Boston, MA, USA; packages: {psych}, {vcdExtra}, {ggplot2}).

## Results

### Subject Characteristics

#### Healthy Subjects

Of the 132 subjects included in the study, 64% (*n* = 84) were female. With a median age of 35 years (range: 18–84 years), most healthy participants were of relatively young age (group 1 [18–35 years]: 50%; group 2 [36–53 years]: 20%; group 3 [54–71 years]: 19%; group 4 [72–89 years]: 11%), right-handed (86%) and had a high educational level (71%).

#### Patients

Ten patients (two females, median age 47 years, range 24–76 years) with normal to moderate word finding skills according to the BIWOS results were included in the clinical pilot part of the study. Most patients were right-handed (80%) and had a high educational level (78%; Table 4).

**Table 4:** Patient characteristics.

**No**	**Gender**	**Age**	**Handedness**	**Education**	**BIWOS** **[raw score (percentile)]**			**Word finding difficulties**
					**Semantic**	**Lexical**	**Overall**	
1	Male	27	Right	UED	63 (69)	31 (16)	47 (42)	Moderate
2	Male	53	Right	UED	95 (>97)	89 (>97)	93 (>97)	Normal
3	Male	27	Left	UED	95 (>97)	97 (>97)	96 (>97)	Normal
4	Male	63	Right	UED equivalent	56 (62)	71 (90)	65 (84)	Slight
5	Male	74	Right	Not reported	63 (69)	59 (73)	61 (69)	Slight
6	Female	34	Right	UED equivalent	83 (96)	64 (82)	73 (88)	Normal
7	Female	76	Left	UED	76 (90)	83 (>97)	79 (96)	Normal
8	Male	40	Right	UED	93 (>97)	91 (>97)	92 (>97)	Normal
9	Male	53	Right	UED	83 (96)	66 (84)	75 (93)	Slight
10	Male	24	Right	UED	81 (95)	73 (92)	77 (95)	Slight

*BIWOS, Bielefeld Screening for word finding difficulties for mild aphasia (29); UED, university entrance diploma*.

### Correctness and Reliability of Object Recognition and Naming

#### Healthy Subjects

Overall, mean correct object recognition and picture naming rates were in the range of 98 ± 4 and 97 ± 4% and were significantly higher in the second as compared to the first run (object recognition: 98.3 ± 3.6 vs. 97.9 ± 4.0%, *p* < 0.001; object naming: 97.7 ± 3.9 vs. 97.2 ± 4.3%, *p* < 0.0001; Table 5). Of note, the rate of delays decreased from the first to the second run (*p* = 0.001), whereas no significant differences between runs were observed for the other error categories (Table 6). However, the overall reproducibility of object naming in-between both runs was excellent, as expressed by an overall Goodman and Kruskal's GK-gamma correlation coefficient of 0.95 ± 0.004 [confidence interval: 0.95; 0.96] (Table 5). The two most common error categories were wrong item naming (43% of all errors) and delay (25%; Table 6).

**Table 5:** Object recognition rates and naming reliability by stimulus.

**A**				**B**				**C**				**D**			
**(one syllable, high WF)**				**(two syllables, high WF)**				**(one syllable, low WF)**				**(two syllables, low WF)**			
**Object**	**Correct object recognition (naming) in %**		**γ [CI]**	**Object**	**Correct object recognition (naming) in %**		**γ [CI]**	**Object**	**Correct object recognition (naming) in %**		**γ [CI]**	**Object**	**Correct object recognition (naming) in %**		**γ [CI]**
	**Run 1**	**Run 2**			**Run 1**	**Run 2**			**Run 1**	**Run 2**			**Run 1**	**Run 2**	
Arm	97 (96)	93 (92)	0.94 [0.85;1]	Eye	100	100		Lightning	98 (96)	98 (96)	1.0 [1;1]	Apple	100	100	
Bank	95	100		Car	99 (98)	100		Bus	99 (96)	99 (98)	0.99 [0.98;1]	Pear	100	98	
Tree	100	100		Glasses	100	100		Egg	98	100		Flower	100	100	
Bed	100	99		Angel	99 (98)	99 (98)	0.93 [0.83;1]	Barrel	95 (92)	97 (96)	0.91 [0.80;1]	Brush	96	97	0.72 [0.30;1]
Bread	98 (95)	99 (98)	0.91 [0.67;1]	Feather	97	99	1.0 [1;1]	Frog	98	99	0.94 [0.78;1]	Dragon	92 (91)	93 (92)	0.95 [0.89;1]
Book	99	100		Window	92	98		Comb	100	100		Bucket	100	100	
Fish	100	100		Finger	92 (88)	97 (95)	0.96 [0.91;1]	Button	94 (93)	96	0.98 [0.94;1]	Fork	98	100	
Foot	100	99 (98)		Trousers	100	100		Crane	98	98	0.83 [0.43;1]	Bell	99	100	
Glass	97 (95)	100 (99)	0.94 [0.88;1]	Island	93 (92)	95	0.98 [0.95;1]	Mouse	98	100		Harp	92	93	1.0 [1;1]
Hand	100	100		Chain	98 (97)	97 (96)	0.76 [0.5;1]	Arrow	98 (97)	97 (95)	0.94 [0.85;1]	Rabbit	95	95	0.96 [0.90;1]
House	99	99		Church	98 (97)	99 (98)	0.99 [0.97;1]	Mushroom	100 (99)	100		Hedgehog	100	100	
Heart	100	99		Suitcase	100	100		Skirt	94	95	0.92 [0.82;1]	Cheese	99 (96)	99 (97)	1.0 [1;1]
Dog	99	100	1.0 [1;1]	King	97	98	1.0 [0.98;1]	Scarf	97	99	0.95 [0.85;1]	Cat	100	100	
Hat	100	100		Crown	100	98		Shoe	100	98		Candle	100	100	
Dress	99	99	1.0 [1;1]	Ladder	100	100		Sponge	92	93	0.95 [0.87;1]	Spoon	100	100	
Cow	97	95 (94)	0.94 [0.85;1]	Lion	98	100		Pig	98 (95)	99	0.97 [0.92;1]	Knife	100 (98)	100 (98)	0.94 [0.78;1]
Mouth	99 (89)	100 (90)	0.87 [0.72;1]	Wall	98 (97)	98	0.98 [0.92;1]	Sword	90 (89)	87 (87)	0.91 [0.81;1]	Mussel	95	95	0.93 [0.83;1]
Horse	100	100		Snake	99	100		Ski	97 (95)	97 (95)	0.91 [0.81;1]	Doll	88	93	0.92 [0.82;1]
Wheel	98 (96)	98 (95)	0.83 [0.56;1]	Key	100	98		Stork	97 (96)	96	0.93 [0.84;1]	Saw	98 (96)	99 (98)	0.83 [0.57;1]
Padlock	100 (98)	100 (98)	0.91 [0.67;1]	Sun	99	100		Pot	99	99	0.98 [0.94;1]	Swing	99	99	0.96 [0.83;1]
Star	100	98		Carpet	100 (98)	100 (99)	0.97 [0.88;1]	Sausage	98	99 (98)	0.99 [0.97;1]	Scissor	100	99	
Chair	99	98		Devil	95	95 (94)	0.92 [0.82;1]	Tooth	99	100		Bow	94 (93)	95	0.98 [0.93;1]
Table	100	99		Drum	100	100		Fence	98 (95)	98 (96)	0.88 [0.73;1]	Syringe	98	98 (97)	0.95 [0.85;1]
Door	99	99		Bird	100 (98)	100 (95)	0.89 [0.68;1]	Tent	99	99	1.0 [1;1]	Alarm clock	86 (84)	92 (91)	0.89 [0.79;099]
Clock	100 (99)	100 (99)	1.0 [1;1]	Newspaper	94 (93)	93	0.99 [0.97;1]	Dwarf	95 (91)	94 (88)	0.95 [0.89;1]	Dice	100	100	
**Overall**	**99** **±** **1 (98 ± 3)**	**99** **±** **2** **(98 ± 3)**	**0.94 [0.91;0.97]**	**Overall**	**98** **±** **3 (97 ± 3)**	**99** **±** **2** **(98 ± 2)**	**0.95 [0.93;0.97]**	**Overall**	**97** **±** **3 (96 ± 3)**	**97** **±** **3**	**0.95 [0.94;0.97]**	**Overall**	**97** **±** **4**	**98** **±** **3** **(97 ± 3)**	**0.96 [0.94;0.97]**

*Object names are given in English (for original German words, please see Table 2). Correct object naming rates are provided in brackets following the correct object recognition rates if differing from those. The percentage of delayed (however correct) object namings was maximum 5.3% and is indicated by colour-encoding for each run: white = no delay; light yellow = <1% delays; yellow = 1–3% delays; orange = >3% delays. Reliability measures (GK-gamma) are provided for each word and overall, including the confidence interval. Light grey: GK-gamma could not be calculated due to perfect object naming in at least one run. Dark grey: Confidence interval was zero due to very low number of errors in at least one run; thus, the respective GK-gamma values were not considered for further analysis. γ, GK-gamma; CI, confidence interval*.

**Table 6:** Error frequencies by category and stimulus class.

**Error**		**Run**	**Stimulus class**				**Overall**
**Category**	**Description**		**A** ** (1 syllable, high WF)**	**B** ** (2 syllables, high WF)**	**C** ** (1 syllable, low WF)**	**D** ** (2 syllables, low WF)**	
2	Delay	1	12.3% (0.2%)	32.3% (1.2%)	25.7% (1.4%)	31.2% (1.5%)	27.2% (1.1%)
		2	12.5% (0.2%)	26.8% (0.7%)	23.1% (1.0%)	27.4% (0.9%)	23.4% (0.7%)
		Pooled	12.4% (0.2%)	30.1% (0.9%)	24.5% (1.2%)	29.6% (1.2%)	25.5% (0.9%)
3	Alternative naming, e.g., dialect-related variant	1	41.5% (0.8%)	16.1% (0.6%)	21.7% (1.2%)	8.4% (0.4%)	18.9% (0.7%)
		2	39.1% (0.8%)	19.5% (0.5%)	21.0% (0.9%)	9.7% (0.3%)	20.4% (0.6%)
		Pooled	40.3% (0.8%)	17.5% (0.5%)	21.4% (1.0%)	9.0% (0.4%)	19.6% (0.7%)
4	Self-corrected	1	16.9% (0.3%)	13.7% (0.5%)	8.0% (0.4%)	10.4% (0.5%)	11.2% (0.4%)
		2	14.1% (0.3%)	18.3% (0.5%)	6.3% (0.3%)	12.4% (0.4%)	11.7% (0.4%)
		Pooled	15.5% (0.3%)	15.5% (0.5%)	7.2% (0.3%)	11.2% (0.5%)	11.4% (0.4%)
5	Wrong	1	29.2% (0.6%)	37.9% (1.4%)	44.6% (2.4%)	50.0% (2.3%)	42.7% (1.7%)
		2	34.4% (0.7%)	35.4% (0.9%)	49.7% (2.2%)	50.4% (1.7%)	44.5% (1.4%)
		Pooled	31.8% (0.6%)	36.5% (1.2%)	46.9% (2.3%)	50.2% (2.0%)	43.5% (1.5%)

*Percentages of error rates are shown relative to the total amount or errors and relative to all stimuli (in brackets). For a more comprehensive description of the error types, please consider Table 3. For a descriptive overview of the delay rates by stimulus/word, cf. Table 5 (colour-encoding). WF, word frequency*.

##### Influence of Word Characteristics on Object Naming Correctness and Reliability

**Figure 2:**
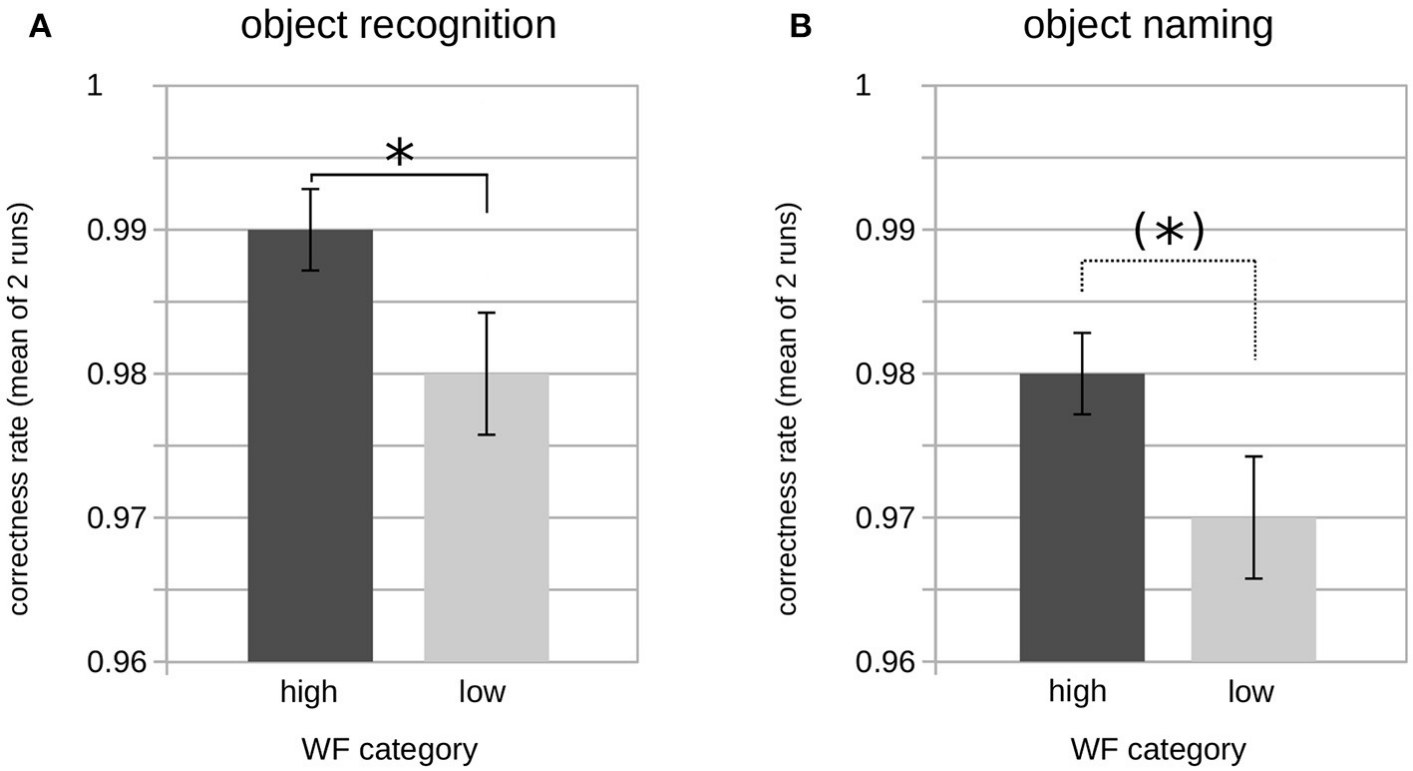
Rates of correct object recognition and naming by word frequency category. Bar plot showing the average correct object recognition **(A)** and object naming **(B)** rates of both runs (pooled) by word frequency (WF). Significant differences according to groupwise *post-hoc* comparison of means are indicated by asterisks [(*)*p* < 0.1; *p < 0.05]. Please note the limited ranges of the *y*-axis, for better readability.

A two-factorial ANOVA including the factors SYLLABLES (two levels: one, two) and FREQUENCY (two levels: high, low) revealed no influence of both factors on the GK-gamma coefficients as a measure of reproducibility or an interaction between them (Table 7). In contrast, a significant main effect was found for the factor FREQUENCY on the correct object recognition rates (*F*_1,96_ = 6.471; *p* < 0.05) as well as on the correct picture naming rates (*F*_1,96_ = 4.166; *p* < 0.05) whereas there was no main or interaction effect on the correct object recognition or naming rates of the factor SYLLABLES (Table 7). Accordingly, *post-hoc* tests revealed significantly higher correct object recognition rates for the high vs. low word frequency (98 ± 3 vs. 99 ± 2%; *p* < 0.05) and a concordant statistical trend regarding the correct object naming rates (98 ± 2 vs. 97 ± 3%, *p* = 0.06; Figure 2).

**Table 7:** Influence of word-specific factors on object recognition and naming.

**Factor**	**Object recognition**		**Object naming**		**Df, residuals**
	***F***	***p*/*p* level**	***F***	***p*/*p* level**	
**Main effects**					
Syllables	0.503	0.480	0.020	0.888	1,96
Word frequency	6.471	0.013	4.166	0.044	
**Interactions**					
Syllables:word frequency	0.920	0.340	0.971	0.327	1,96

*F statistics and p-values are provided for the two dependent variables object recognition and object naming*.

*Post*-*hoc* comparisons revealed the lowest rates of delays for word class A (high WF, one syllable) as compared to all other classes (*p* < 0.0001, FDR-corrected, Table 6). In contrast, category III responses (e.g., dialect-related variants; see Table 3 and Supplementary Table 2) were more frequent when naming one-syllable words and were highest in word class C (C-B: *p* < 0.01; C-D: *p* < 0.001; A-D: *p* < 0.01, FDR-corrected; Table 6). Self-corrections were equally distributed across the stimulus classes. Of note, all unexpected correct naming alternatives (e.g., dialect variants) encountered in the study are provided in the supplement (Supplementary Table 2). According to our hypothesis, the rate of wrong object namings increased with the difficulty level and was particularly more frequent in the stimulus classes of low WF (A-B: *p* < 0.01; A-CD: *p* < 0.0001; B-C: *p* < 0.001; B-D: *p* < 0.05, FDR-corrected; Table 6).

##### Influence of Subject Characteristics on Object Naming Correctness

To analyze the influence of subject characteristics on correct object recognition and naming rates (sum of both runs), we performed a three-factorial ANOVA with the factors GENDER (two levels), EDUCATION (two levels) and AGE GROUP (four levels). We, here, found a significant main effect of the factors AGE GROUP and EDUCATION on both correct object recognition and naming rates as well as a significant interaction between those two factors (Table 8). In contrast, the factor GENDER had no significant main effect on either object recognition or naming correctness and showed no interactions regarding the dependent variable object naming correctness. However, we observed an interaction with the factor AGE GROUP when analysing the effects on object recognition correctness (Table 8).

**Table 8:** Influence of subject-specific factors on object recognition and naming.

**Factor**	**Object recognition**		**Object naming**		**Df, residuals**
	***F***	***p*/*p* level**	***F***	***p*/*p* level**	
**Main effects**					
Age group	39.8	<0.0001	43.5	<0.0001	1,121
Education	4.4	0.039	4.2	0.044	
Gender	1.7	0.193	0.6	0.424	
**Interactions**					
Age group:education	11.1	0.001	9.1	0.003	1,121
Age group:gender	4.4	0.037	2.7	0.105	
Education:gender	0.05	0.830	0.0	0.981	
Age group:education:gender	4.0	0.048	1.7	0.189	

*F statistics and p-values are provided for the two dependent variables object recognition and object naming*.

**Figure 3:**
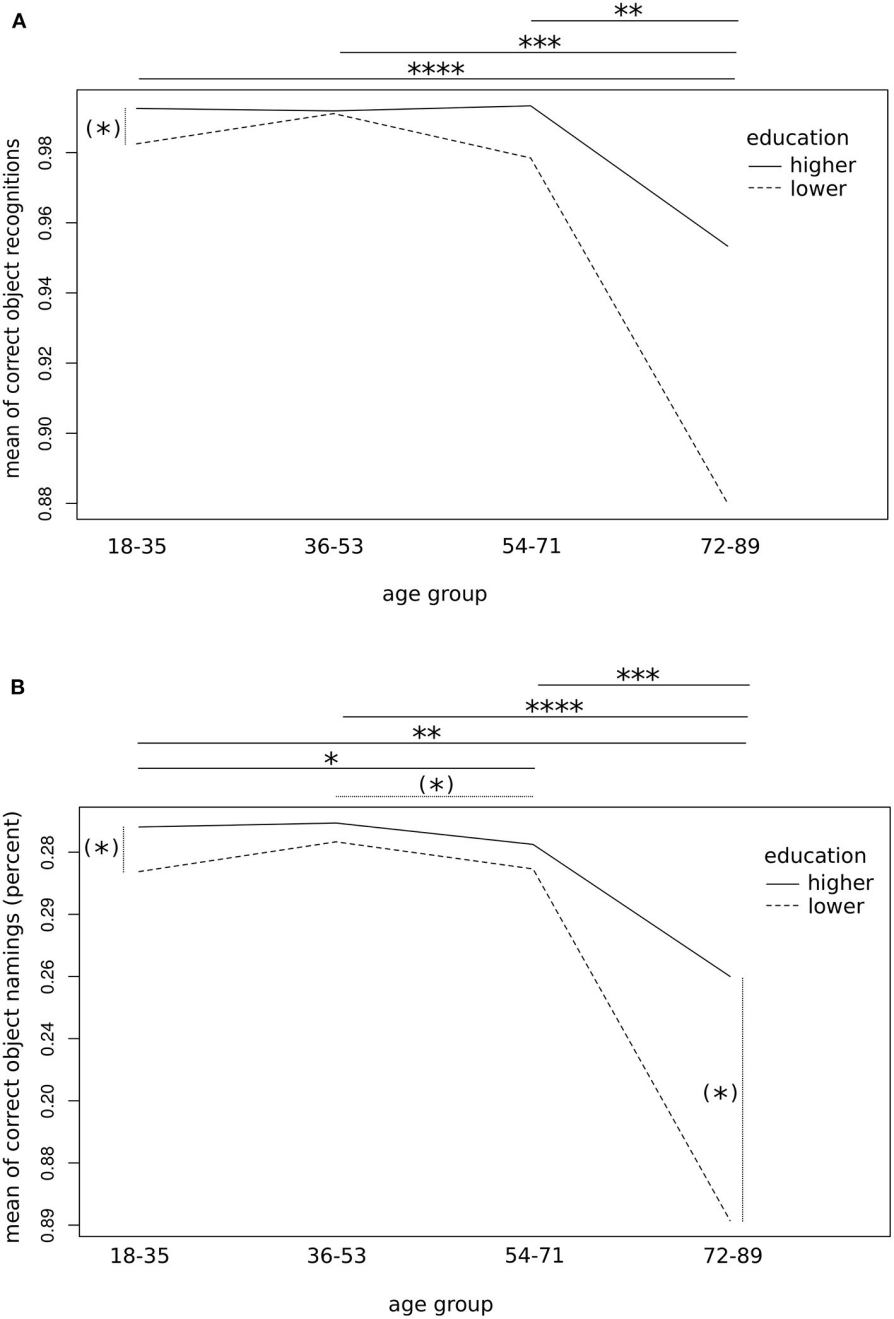
Influence of age group on object naming performance. Interaction plots showing average correctness of object recognition **(A)** and naming **(B)** by age groups and education level (i.e., with/without university admission diploma or equivalent). Pooled data of both runs per 100 trials are provided. Significant differences according to groupwise *post-hoc* comparison of means followed by FDR correction are indicated by asterisks [(*)*p* < 0.1; *p < 0.05; **p < 0.01, ***p < 0.001, ****p < 0.0001]. Please note the limited ranges of the *y*-axis, for better readability.

Second-level one-factorial ANOVA confirmed a significant main effect of the factor AGE in both the subgroups of lower and high education levels on the correct object recognition rates (low: *F*_1,35_ = 12.3, *p* < 0.01; high: *F*_1,90_ = 12.2, *p* < 0.001) as well as on the correct object naming rates (low: *F*_1,35_ = 12.3, *p* < 0.01; high: *F*_1,90_ = 17.0, *p* < 0.0001), thus suggesting the strongest influence of age on object naming in highly educated subjects. Of interest, *post-hoc* tests revealed a significantly lower rate of correct recognition as well as object naming for elderly subjects (age group 4) compared to all other age groups (*p* < 0.01, FDR-corrected; Figure 3). In addition, subjects of slightly advanced age, i.e., between the age of 54 and 71 years showed similar object recognition performance (*p* > 0.1) but worse object naming rates compared to younger individuals (age group 3 vs. 1 [2]: *p* < 0.05 [*p* = 0.07], FDR-corrected; Figure 3). These findings go along with a larger variance and less skewed data distribution in the elderly—particularly when less educated—as compared to young age (Supplementary Figures 1, 2).

In contrast, analysed by age categories, a one-factorial ANOVA showed a significant main effect of the factor EDUCATION on the picture naming performance only for young subjects that represented the largest age group (object recognition: *F*_1,64_ = 4.0, *p* < 0.05; object naming: *F*_1,64_ = 5.0, *p* < 0.05). No noteworthy effect of this factor was found in the other groups, apart from the elderly group, which showed a statistical trend (object naming: *F*_1,11_ = 3.4, *p* = 0.09). *Post*-*hoc* tests confirmed a statistical trend towards better picture recognition and naming for the subgroups of young and elderly subjects (*p* = 0.09, FDR-corrected; Figure 3). In summary, the effect of the subject's age—and particularly the affiliation to the age group of 72 or more years—seems to overweigh clearly the effect of the educational level on correct object identification and naming.

##### Influence of Subject Characteristics on Object Naming Reliability

In accordance with the factors on naming performance, we here analysed the influence of subject characteristics on the retest reliability of the object naming, i.e., on GK-gamma coefficients using a three-factorial ANOVA that included the factors GENDER (two levels), EDUCATION (two levels) and AGE GROUP (four levels).

In line with our results regarding object recognition and naming correctness, the factor AGE GROUP had a significant main effect on naming reliability (*F*_1,93_ = 5.3, *p* < 0.05). However, no main effect was found for the factors EDUCATION and GENDER. Although no two-way interactions were observed, the ANOVA revealed a significant interaction between the factors AGE GROUP × EDUCATION × GENDER (*F*_1,93_ = 6.0, *p* < 0.05).

**Figure 4:**
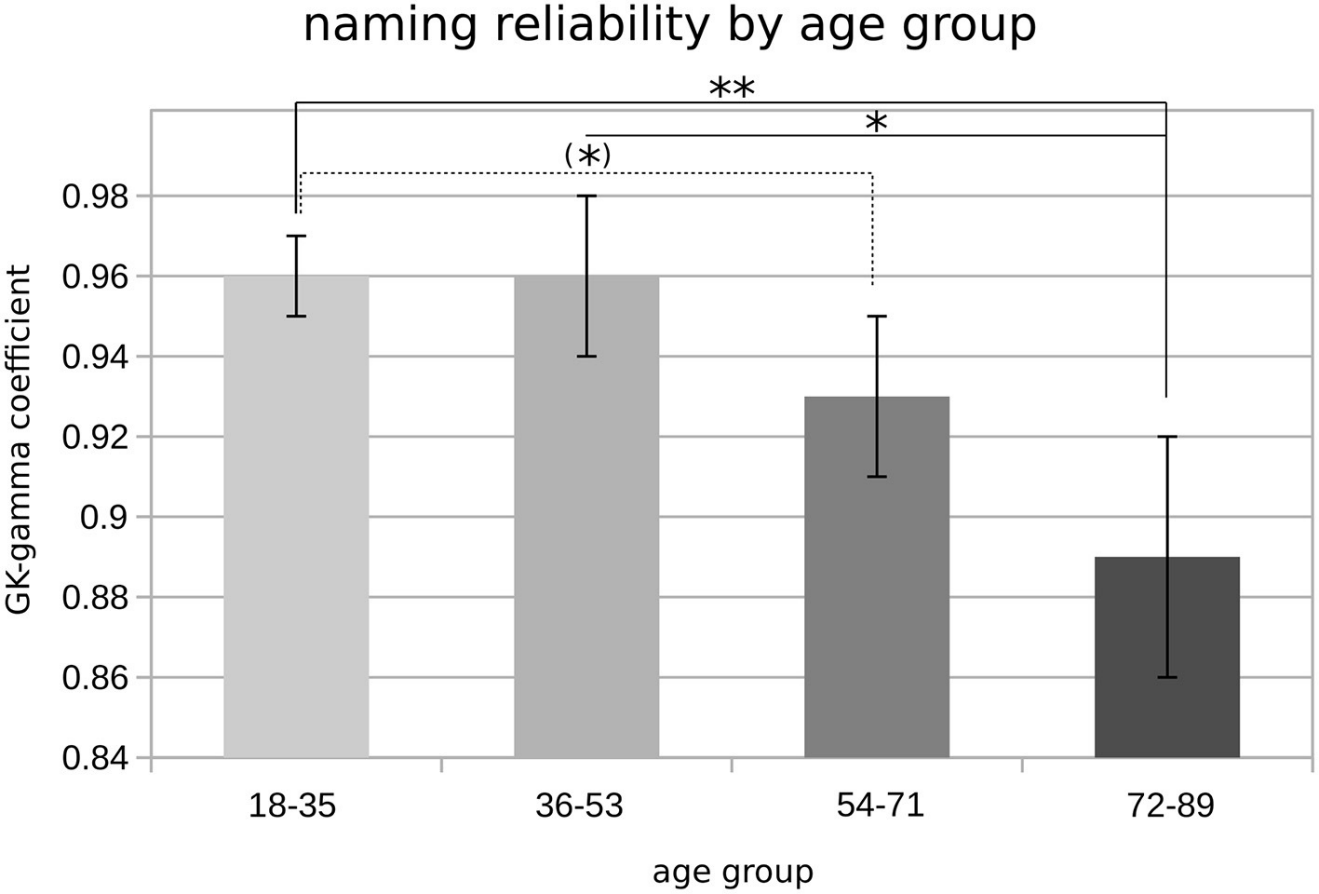
Object naming reliability by age groups. Bar plot showing mean GK-gamma coefficients (*y*-axis), grouped by age categories; error bars represent SEM; significance levels are indicated by asterisks [(*)*p* < 0.1; *p < 0.05; **p < 0.01].

*Post-hoc* tests showed that higher age was associated with worse test–retest reliability of the naming responses. Accordingly, lower GK-gamma coefficients were found in the age group of 72 years or older as compared to subjects younger than 54 years (i.e., groups 1 and 2; *p* < 0.05, FDR-corrected; Figure 4).

#### Patients

In the pilot cohort of patients, showing evidence for impaired lexicosemantic word finding skills according to the BIWOS score in at least half of the cases, results for correct object recognition and naming (Table 9) were not significantly different from those of an age-matched cohort of *n* = 30 healthy subjects (median age [range] = 46 [24;79] years; 40% male; pooled object naming [recognition] rate: 96.4 ± 4.9 [97.4 ± 4.2]%; *p* > 0.1). Overall, the test–retest reliability of the response category (five levels; see Table 3) was high, as expressed by a GK-gamma coefficient ranging from 0.74 (A) to 0.94 (D; Table 9). In line with our results from the healthy population indicating high age (above 72 years) as the major factor influencing task performance, we here observed the least correct object recognition and naming rates in the two older patients (patient 5: 91/88%; patient 7: 94/91%). In contrast to the healthy subjects, the better object recognition performance in run 2 could not be reproduced in the patients (run 1: 97 ± 2/97 ± 3% vs. run 2: 97 ± 4/95 ± 5%; *p* > 0.1). However, we found a significantly lower frequency of delayed responses in the second run (run 1: 5.4 ± 7.7 vs. run 2: 2.7 ± 5.7%; *p* < 0.001; Table 9). There was no significant correlation of correctness, delay or reliability of object identification or naming with the clinical aphasia score (BIWOS).

**Table 9:** Object naming performance of patients by stimulus class and run.

	**Run**	**Stimulus class**				**Overall**
		**A**	**B**	**C**	**D**	
Correct object recognition (/naming) rates	1	98 ± 2% (97 ± 3%)	97 ± 4%(96 ± 5%)	98 ± 3%(97 ± 4%)	96 ± 4%	97 ± 3% (96 ± 4%)
	2	96 ± 4%(95 ± 6%)	98 ± 4%(97 ± 5%)	97 ± 4%(95 ± 8%)	95 ± 5%	97 ± 4% (95 ± 6%)
	Pooled	97 ± 3%(96 ± 4%)	98 ± 4% (97 ± 5%)	97 ± 3% (96 ± 6%)	95 ± 5%	97 ± 4% (96 ± 5%)
Reliability (GK-gamma [CI])		0.74 ± 0.10 [0.54; 0.95]	0.83 ± 0.08[0.67; 0.99]	0.87 ± 0.06 [0.76; 0.97]	0.94 ± 0.03[0.88; 1.00]	0.86 ± 0.03 [0.80; 0.92]

*Correct object recognition and naming rates as well as GK-gamma coefficients are indicated by stimulus class, run and overall. Please note that correct object naming rates are indicated in brackets following the corresponding object recognition rates if different from those. The average percentage of delayed (however correct) object namings per word class was maximum 7.2% and is indicated by colour-encoding for each run: yellow = <3% delays; orange = 3–5% delays; light red = >5% delays*.

## Discussion

This work provides the first freely available data set of pictures, developed for experimental and clinical use (e.g., in the context of pre-surgical and intraoperative functional language mapping), specifically for German-speaking subjects. The CoNaT was especially designed for the context of language mapping using picture naming, where highly reliable naming performance is a pre-requisite of successful testing. The picture set, consisting of 100 black and white drawings, stratified by word length (number of syllables) and word frequency, showed excellent correct object recognition and naming rates as well as high reliability coefficients across all item categories and subjects. However, a small learning effect was observed across the two runs of the test. Moreover, we found a significant negative effect of low word frequency and high age (older than 72 years) on the task performance.

### Influence of Subject Characteristics

Amongst subject-related factors, age had the strongest effect on both the picture naming correctness and the test–retest reliability of the naming responses. Its negative effect on the task performance increased with age and was most evident in elderly subjects who are 72 years or older. The high effect size of the factor AGE was also reflected by its significant correlation with the object naming performance in the patient cohort, despite the small sample size of *n* = 10.

This finding is widely in line with previous research that also found an effect of age on language skills in general and picture naming in particular (36–38). Furthermore, multiple subject-related factors including vision impairment, general cognitive decline, reduced attention span, slowed perceptual analysis (37, 39, 40), as well as linguistic factors such as weakening of semantic connections within the language system (36, 41) have been discussed to affect language performance.

In line with previous publications of other groups (42, 43), a high general educational level (i.e., qualification for admission to university or equivalent) was associated with higher rates of correct picture recognition and naming in our data set. This effect was most prominent in the subgroups of elderly participants (i.e., 54 years or older) for which the factor education was more balanced as opposed to the mostly highly educated younger participants (cf. Limitations). The finding, however, that educational level did not correlate with the test–retest reliability of the responses, might reflect the robustness of the factorial influence on naming correctness, independent of supposable learning effects between both runs.

From the clinical point of view, the clearly impaired and less reliable task performance of elderly healthy subjects, especially when their level of education is low, points out that language mapping and monitoring results should be interpreted with particular caution to avoid false-positive results. In such cases, a more rigorous selection of the items to be included in the picture set prior to the clinical use might be advisable to reduce the risk of misinterpretations, e.g., by omitting items with generally suboptimal correct naming rates and delayed responses (cf. Table 5). Moreover, increasing the usual number of individual test runs may be helpful to make sure that potentially problematic items are excluded.

As opposed to age and educational level, we found no significant influence of the factor GENDER on picture naming correctness, indicating that the selected items can be considered gender neutral and appropriate for testing procedures with both male and female participants. This finding could explain the disagreement, e.g., with the previous work of (42) who reported a gender effect with mostly better performance of male subjects in a picture naming task, which they explained by specific components of their picture set [e.g., items like “tripod,” “compass,” and “dart”; cf. Table 3 in (42)]. In this regard, the result of “gender neutrality” met with our expectations, given that we excluded words with assumed gender effect, e.g., “screw-driver” from our picture set a priori in order to establish a robust, gender-independent picture set for clinical use.

The robustness of the picture set is also reflected by the overall excellent and highly reliable picture naming performance of the patient cohort, showing no significant difference in naming correctness rates compared to a matched group of healthy subjects. At least for the tested cohort of patients with utmost mild aphasic symptoms, we also found no significant correlation between picture naming correctness and lexicosemantic performance according to the formal testing using the BIWOS. This finding underlines the intention of the picture set, which was not designed to be used as a sensitive screening instrument for (even mild) aphasia but rather as reliable and robust monitoring tool, also suited for patients with mild aphasic symptoms.

### Influence of Word Characteristics

#### Response Correctness

In this study, we investigated the influence of two important word characteristics, i.e., the word frequency and the number of syllables, on the correctness of picture recognition and verbal naming responses. Here, we used the factor lexical word frequency as the most common and standardised measure of frequency of (word) use in everyday life. We found a significantly better performance, i.e., higher correct object recognition and naming rates, when the subjects were asked to name high-frequency words. In addition, there were fewer delays when naming high-frequency words, at least in the subset of monosyllables. These results agree well with previous research that also showed an effect of word frequency on naming accuracy [e.g., (44–46)].

In contrast to the word frequency, there was no significant influence of the factor word length, expressed by the number of syllables (mono- vs. bisyllabic), on neither the correctness of picture recognition or naming nor the retest reliability of the naming responses between the two runs. This finding is in line with the results of Santiago et al. (47) who also did not find a significant influence of the number of syllables (also comparing mono- vs. bisyllabic words) on the occurrence of errors in a standard picture naming task.

#### Delay

We observed significantly less delays (as a measure of response latency) for class A words (i.e., monosyllabic, high WF) as compared to all other word categories.

This finding indicates an influence of word frequency on response latency only for monosyllabic words and, vice versa, an influence of the number of syllables only for high-frequency words, thereby reflecting the heterogeneous results of previous studies regarding the effect of word length and word frequency on response latency. In line with others (46, 48), Alario et al. (49) identified word frequency but not the number of syllables as significant contributors for the prediction of response latency. Other research groups, in contrast, could not confirm an effect of word length on response latency (50–52).

The divergent study results could be explained by methodological differences across studies such as the distinct characteristics of the applied picture sets. For instance, we here used comparatively high median word frequencies and a small range of word lengths (number of syllables), due to the primary objective of our study to develop a robust language monitoring tool rather than a very sensitive screening instrument. Further possible influencing factors include (i) the different age ranges of the study participants (usually university students younger than 30 year-old compared to a wide age range of 18–89 years in our study), (ii) interactions with other item- or word-related characteristics [e.g., lexical/conceptual characteristics such as age of acquisition, animacy, relevance to everyday life, frequency of syllables or word form characteristics such as phonological or morphological complexity; cf. (49, 53, 54)], and (iii) priming processes (55, 56) inherent to the respective picture sets, which were not controlled in this study (see also Limitations).

Taken together, due to the influence of word characteristics on both naming correctness and response latency, it might be advisable to start the clinical testing routine for patients with relatively advanced aphasic symptoms using the components of the stimulus classes A–D consecutively in alphabetic order. Items might even be omitted class-wise in severe cases.

### Alternative Naming Variants and Clinical Implications

In addition to different response delay rates between mono- vs. bisyllabic high-frequency words, the word-class-wise analysis also showed a higher rate of unexpected, alternative responses like over-specifications, dialectal or cultural variants for monosyllabic words. In accordance with our hypothesis that rather short, monosyllabic words are generally more prone to over-specification (e.g., “water glass” for “glass”), this was the reason for two thirds of the unexpected alternative responses in word class A in our study.

In clinical practise, e.g., for monitoring during awake surgery using DCS or for preoperative language mapping using TMS, where robustness of the test is of particular importance to assure correct identification of transient language impairments, it might be advisable to reduce the pictures by avoiding items with relatively high alternative naming rates (cf. Supplementary Table 2). However, given the overall excellent reliability of the naming responses as expressed by high GK-gamma coefficients (cf. Table 5), alternative naming responses should usually be identifiable in the preparatory test run, i.e., the baseline investigation, which allows to tailor the picture set on an individual basis (cf. Influence of Subject Characteristics section). In general, a baseline investigation of naming performance is highly recommended, especially regarding the clinical application in patients using TMS and/or DCS for language mapping, in order to identify speech language difficulties such as increased response latencies (delayed naming) related to distinct stimuli/words.

### Learning Effect

Although the overall reproducibility of the object naming in-between both runs was excellent (GK-gamma = 0.95), the mean correct object recognition and naming rates improved slightly from the first to the second run in the healthy volunteers. In line with this finding, we found a concordant decrease in the rate of delayed namings. These findings might result from a repetition priming effect, which is considered an implicit learning phenomenon of non-hippocampal origin described for repeated picture naming, correlating to reduced neural activity in repeated conditions [e.g., (57)], which lasts for at least several weeks [cf. (58) for review]. In this regard, the observation of a learning effect further supports the evidence that high word frequency (as a measure of repetition) correlates with better picture naming performance.

In contrast to healthy subjects, the second run was not associated with a higher overall rate of correct object naming or recognition in patients, which could be attributed to the much smaller sample size as well as to the comparatively stronger effects of reduced attention, lower cognitive resilience or exertion fatigue in this cohort [cf. (59–61)]. However, repetition had a significant and—compared to the healthy subjects—relatively strong facilitating effect on the rate of delayed namings in this cohort. This finding indicates that naming delays are particularly prone to repetition priming effects in patients. Accordingly, our data support the assumption that the risk of spontaneous naming errors, unrelated to TMS/DCS stimulation, decreases with the number of repetitions. On the other hand, it seems likely that the susceptibility to TMS interference expressed by naming errors in general and by prolonged naming latencies in particular decreases along with the repetition of stimuli during a TMS/DCS mapping. Therefore, it seems mandatory to define an optimal trade-off regarding the size of the stimuli/word set to be used during language mapping, as well as to take the number of stimuli/word repetitions into account when analysing the mapping results. A more detailed investigation of this topic, however, lies beyond the scope of this study and deserves to be further addressed in the future.

### Limitations

As the intended use of the picture set is to serve, i.a., for clinical mapping and monitoring of patients with brain tumours, which mostly occur in advanced age, our study cohort comprises a broad age range—in contrast to the vast majority of previous, similar studies. However, due to several constraints regarding the recruitment of older subjects (i.e., reduced access to the population via existing databases and media, morbidity/reduced mobility impeding on-site participation, non-matching of in- and exclusion criteria), the cohort of older subjects remains underrepresented in our study collective. Moreover, the factorial analysis regarding the influence of age and educational level suffers from an unavoidable interaction between both factors, which we attribute mostly to a considerably increased access to high education over the past decades.

Although we analysed two major word-related factors on picture naming performance and response delay, i.e., word frequency and the number of syllables, other possible factors such as alternative measures of word familiarity [e.g., frequency of syllables and age of acquisition; (53)] and word length as well as picture-related factors like the visual complexity of the drawing, image agreement and imageability [e.g., (49)] were not controlled in this study.

The CoNaT has been specifically designed for German native speakers although the stimuli might be well-suited to be used also in other languages. Please note that the suitability of individual items should be checked prior to the test administration to ensure their fit with respect to relevant linguistic criteria.

## Conclusion

In summary, the CoNaT provides an overall robust and reliable picture naming tool, optimised for the clinical use to map and monitor language functions in patients. We here provide normative data along with practical, clinical suggestions for the administration of the picture set, hereby taking important word- and subject-related factors of object recognition and naming into account. Based on the results, we are convinced that the entire picture set can be readily used in healthy subjects and patients, even with mild to moderate aphasic symptoms but should always be tested and—if necessary—reduced on an individual basis, particularly in elderly subjects of low educational level and patients. Here, starting to test with the most robust stimulus class A (high WF, monosyllables) over B (high WF, bisyllables) to C and D (low WF) and paying particular attention to items that are comparatively prone to alternative naming variants seem to be advisable.

## Data Availability Statement

The raw data supporting the conclusions of this article will be made available by the authors, without undue reservation.

## Ethics Statement

The studies involving human participants were reviewed and approved by local ethics committee (Ethikkommission der Medizinischen Fakultät der Universität zu Köln). Written informed consent for participation was not required for this study in accordance with the national legislation and the institutional requirements.

## Author Contributions

CWL: conceptualisation, methodology, formal analysis, investigation, writing – original draught, visualisation, and supervision. JP: formal analysis, investigation, and writing – original draught. SK: investigation and data curation. CN: investigation and writing – review and editing. CG: conceptualisation and writing – review and editing. RG: funding acquisition and writing – review and editing. KJ: conceptualisation, methodology, writing – original draught, and supervision. All authors contributed to the article and approved the submitted version.

## Conflict of Interest

The authors declare that the research was conducted in the absence of any commercial or financial relationships that could be construed as a potential conflict of interest.
