# Supplementary material for: The Cologne Picture Naming Test for Language Mapping and Monitoring (CoNaT): An Open Set of 100 Black and White Object Drawings
Source: Front Neurol. 2021 Mar 3;12:633068. doi: 10.3389/fneur.2021.633068 (PMC7966504; doi:10.3389/fneur.2021.633068)
Supplement: Supplementary file 1 [file Table_1.pdf]

Supplementary Table S1: Stimuli omitted from original test set. The table provides an overview of the twelve words in German [translated in English] corresponding to the stimuli which were discarded from the original set of n=112 stimuli due to comparatively poor object naming performance in healthy subjects. Decisions to discard the items were primarily based on correct object recognition rates compared to the other stimuli of the respective set (A-D); however, reliability was also considered, with GK-gamma <0.9 being generally regarded less than optimal.

**Object name**	**Syllables**	**WF**	**Correct object recognition rate** (runs 1 & 2 pooled)	**GK-gamma** (γ ± SD [CI]
Anker[$] [anchor]	2	3	88.5 %	0.996 ± 0.01 [0.99;1]
Bogen[§] [bow]	2	14	95.3 %	0.85 ± 0.14 [0.58;1]
Biene[$] [bee]	2	2	87.0 %	0.97 ± 0.03 [0.92;1]
Clown[$] [clown]	1	9	88.5 %	0.96 ± 0.03 [0.89;1]
Foto[$] [photograph]	2	121	71.0 %	0.98 ± 0.02 [0.95;1]
Hai[$] [shark]	1	1	91.0 %	0.91 ± 0.11 [0.58;1]
Kreis [circle]	1	111	91.5 %	0.97 ± 0.03 [0.91;1]
Kreuz[$] [cross]	1	29	83.5 %	0.89 ± 9.96 [0.77;0.999]
Pfau[$] [peacock]	1	3	86.0 %	1 ± 0 [1;1]
Soldat[$] [soldier]	2	24	74.0 %	0.99 ± 0.01 [0.96;1]
Spiegel[$] [mirror]	2	36	89.5 %	0.92 ± 0.05 [0.96;1]
Turm[§] [tower]	1	12	94.3 %	0.86 ± 0.18 [0.51;1]

CI: confidence interval; SD: standard deviation; WF: word frequency in German.

§)discarded after first interim analysis (n=42); $)discarded after second interim analysis (n=100).
