# Supplementary material for: The Cologne Picture Naming Test for Language Mapping and Monitoring (CoNaT): An Open Set of 100 Black and White Object Drawings
Source: Front Neurol. 2021 Mar 3;12:633068. doi: 10.3389/fneur.2021.633068 (PMC7966504; doi:10.3389/fneur.2021.633068)
Supplement: Supplementary file 2 [file Table_2.pdf]

Supplementary Table S2: Unexpected, alternative naming responses, by stimulus class. The percentage of subjects which used alternative words for picture naming (response category III) in at least one trial are provided following the expected word (i.e. overall, third column) and by verbal response (right column). Moreover, the median age and range of the respective subjects are given. For a better overview, alternative namings which were given by 3-5% of the subjects are highlighted in orange, by > 5% of the subjects in red.

class	item	Expected naming: response cat. I/II (total rate of subjects giving alternative naming responses)	Unexpected namings: response cat. III (rate of subjects giving alternative naming responses; median age [age range] of respective subjects)
A	mouth	Mund (17%)	Lippen (17%; 58 [30-83] yrs)
A	bread	Brot (3%)	Brotscheibe (1%; 41 yrs),<br>Brotlaib (2%; 30 [23-36] yrs),<br>Stück Brot (1%; 39 yrs)
A	locker	Schloss (3%)	Vorhängeschloss (3%; 66 [39-79] yrs)
A	wheel	Rad (3%)	Wagenrad (2%; 55 [30-79] yrs),<br>Karrenrad (1%; 59 yrs)
A	cross	Kreuz (2%)	Warnkreuz (2%; 62 [44-79] yrs)
A	clock	Uhr (1%)	Wanduhr (1%; 34 yrs)
A	glass	Glas (1%)	Wasserglas (1%; 28 yrs)
B	bird	Vogel (5%)	Vögelchen (1%; 25 yrs),<br>Spatz (4%; 42 [21-40] yrs),<br>Sperling (1%; 55 yrs)
B	finger	Finger (3%)	Einzelner Finger (1%; 41 yrs),<br>Zeigefinger (1%; 40 yrs),<br>Mittelfinger (2%; 30 [24-36] yrs)
B	chain	Kette (2%)	Kettenglieder (1%; 41 yrs),<br>Eisenkette (1%; 19 yrs)
B	church	Kirche (2%)	Kirchturm (2%; 67 [57-76] yrs),<br>Kapelle (1%; 26 yrs)
B	island	Insel (2%)	Palmeninsel (1%; 79 yrs),<br>Südseeinsel (1%; 79 yrs)
B	angel	Engel (1%)	Schutzengel (1%; 83 yrs)
B	car	Auto (1%)	PKW (1%; 79 yrs)
B	carpet	Teppich (1%)	Läufer (1%; 81 yrs)
B	devil	Teufel (1%)	Satan (1%; 73 yrs)
C	arrow	Pfeil (9%)	Pfeil nach rechts (8%; 41 [24-60] yrs),<br>Richtungspfeil (1%; 73 yrs)
C	pot	Topf (8%)	Kochtopf (3%; 67 [26-79] yrs),<br>Kessel (5%; 54 [26-59] yrs)
C	dwarf	Zwerg (7%)	Gartenzwerg (5%; 41 [24-73] yrs),<br>Heinzelmann/-männchen (2%; 51 [41-61] yrs)
C	bus	Bus (4%)	Omnibus (2%; 76 [59-79] yrs),<br>Autobus (2%; 81 [81;81] yrs)
C	pig	Schwein (3%)	Schweinchen (2%; 28 [25-30] yrs),<br>Sau (2%; 58 [55-60] yrs)
C	barrel	Fass (2%)	Holzfass (1%; 60 yrs),

			Weinfass (1%; 24 yrs)
C	crane	Kran (2%)	Drehkran (1%; 60 yrs),<br>Baukran (1%; 52 yrs)
C	fence	Zaun (2%)	Gatter (1%; 76 yrs),<br>Lattenzaun (2%; 75 [71-79] yrs)
C	sausage	Wurst (1%)	Würstchen (1%; 27 yrs)
D	cheese	Käse (4%)	Stück Käse / Käsestück (4%; 41 [27-81] yrs)
D	knife	Messer (2%)	Buttermesser (1%; 41 yrs),<br>Schmiermesser (1%; 60 yrs)
