# Supplementary material for: The Cologne Picture Naming Test for Language Mapping and Monitoring (CoNaT): An Open Set of 100 Black and White Object Drawings
Source: Front Neurol. 2021 Mar 3;12:633068. doi: 10.3389/fneur.2021.633068 (PMC7966504; doi:10.3389/fneur.2021.633068)
Supplement: Supplementary file 4 [file Data_Sheet_2.pdf]

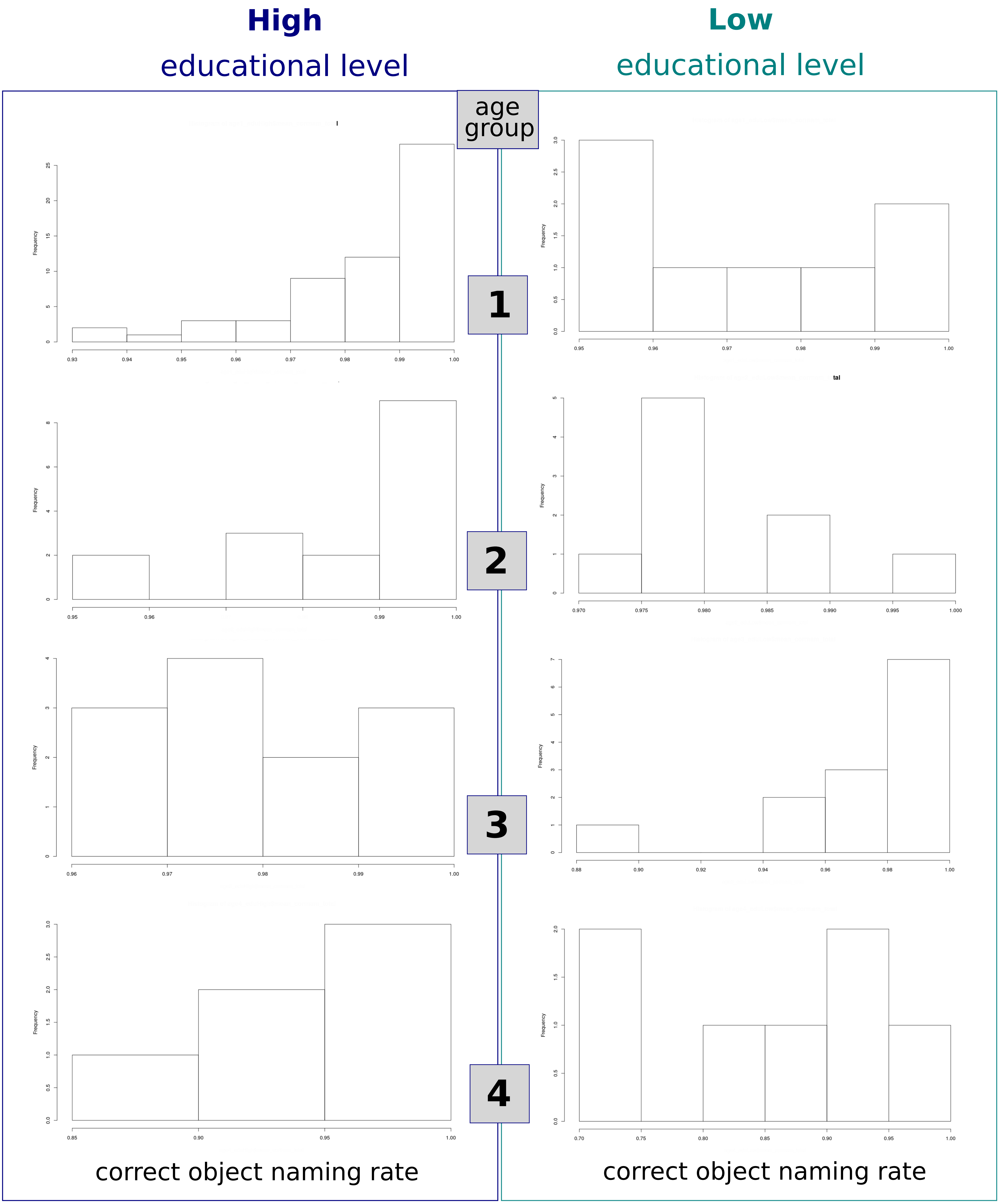

Supplementary Figure S2. Correct object naming by age groups and educational levels. The histograms show the data distribution ranging from a skewed distribution in younger subjects of high educational level to a larger variance with normal data distribution in less educated elderly. Left column: high educational level; right column: low educational level; rows: age categories from 1 (first row) to 4 (last row).
