# Supplementary figures and images for: The Cologne Picture Naming Test for Language Mapping and Monitoring (CoNaT): An Open Set of 100 Black and White Object Drawings
Source: Front Neurol. 2021 Mar 3;12:633068. doi: 10.3389/fneur.2021.633068 (PMC7966504; doi:10.3389/fneur.2021.633068)

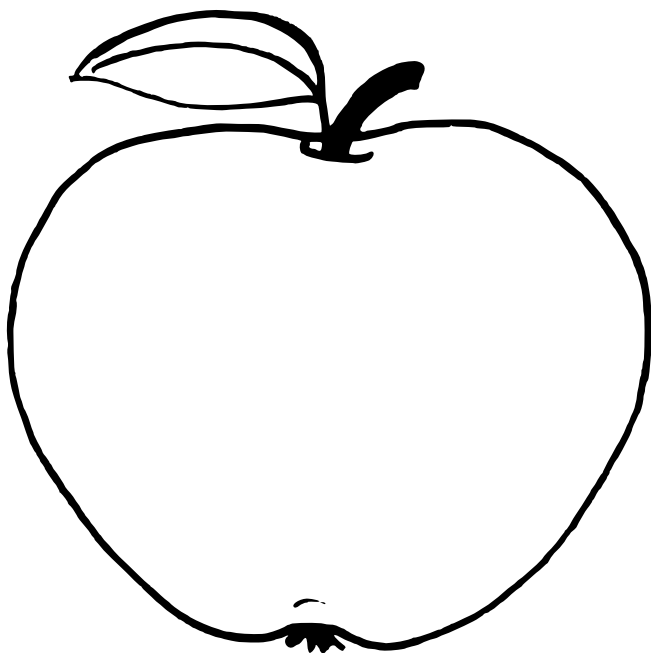

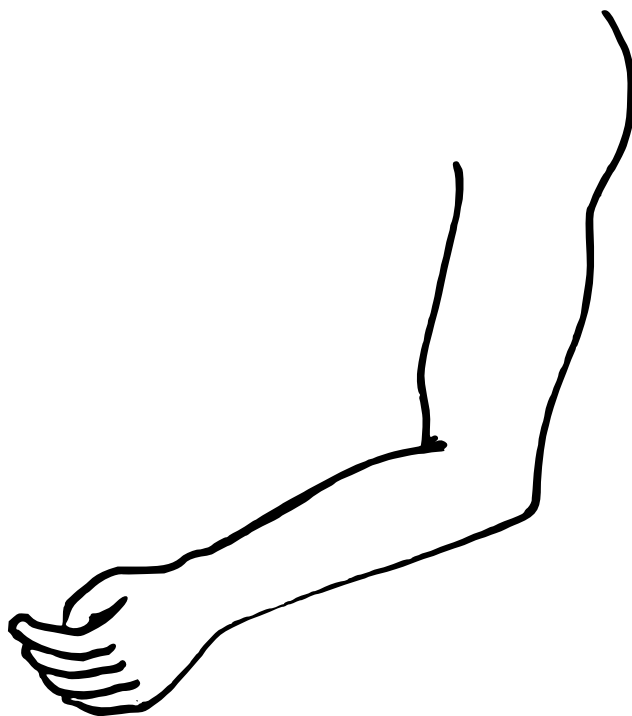

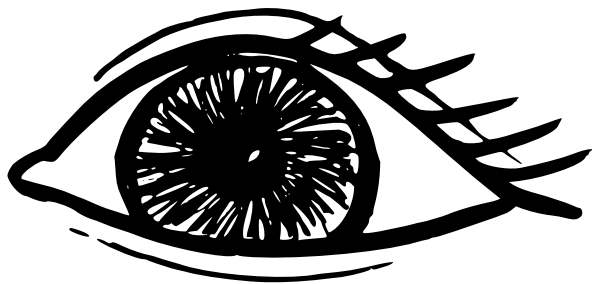

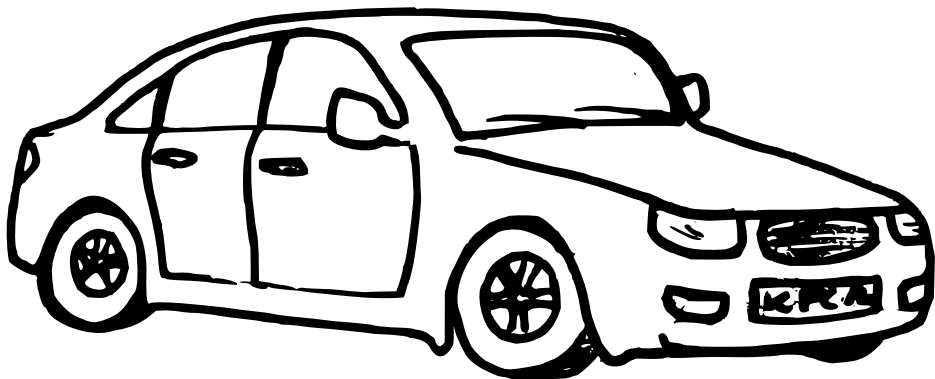

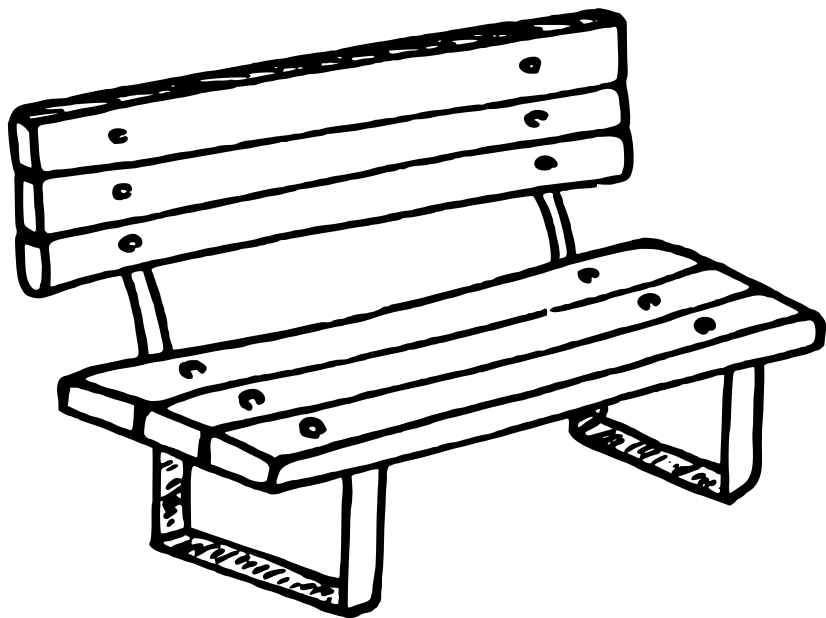

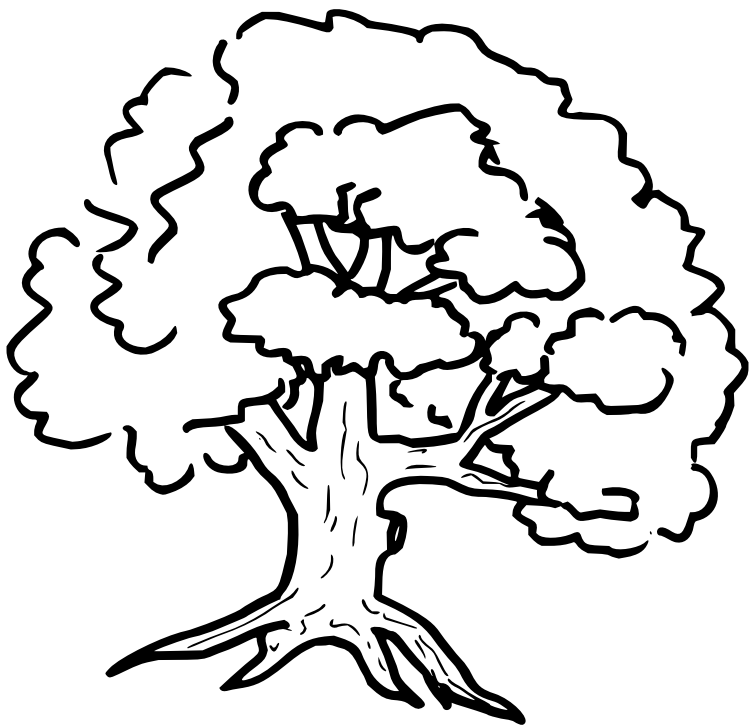

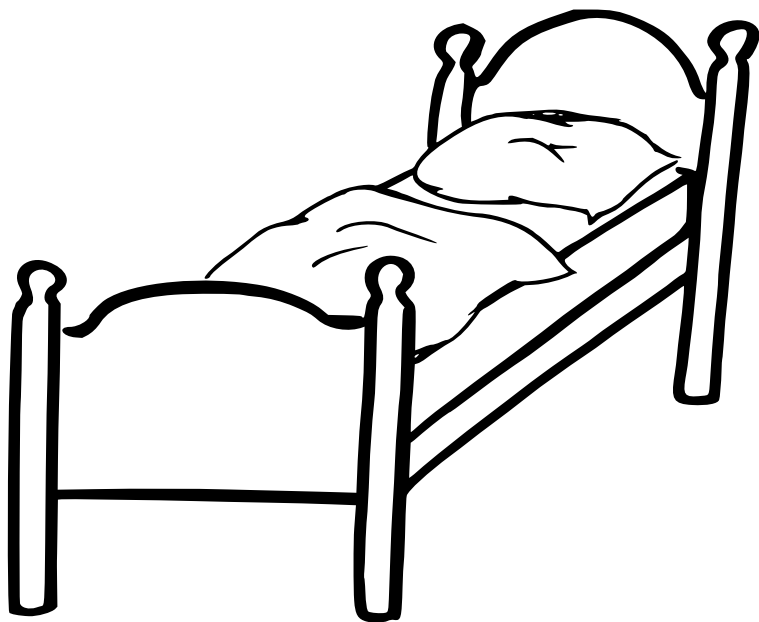

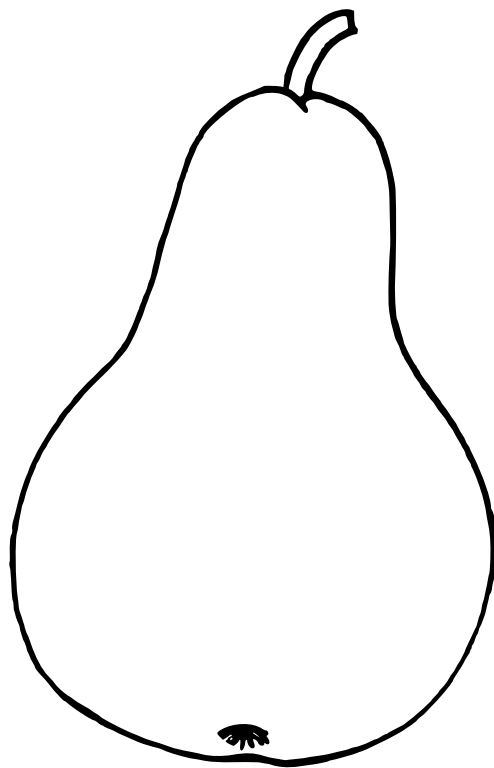

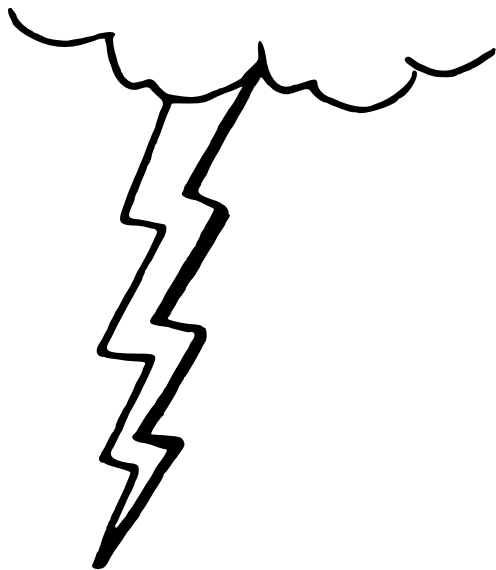

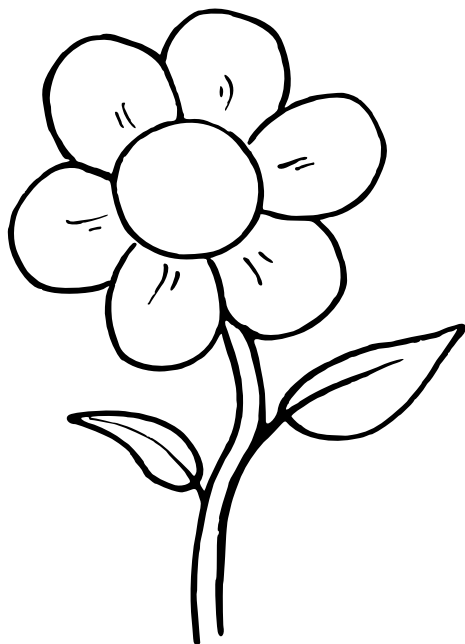

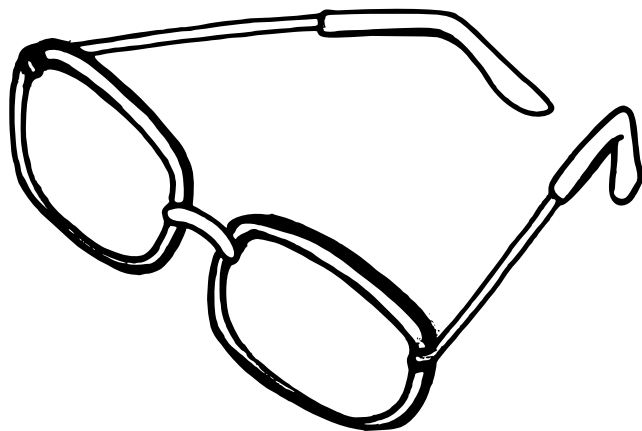

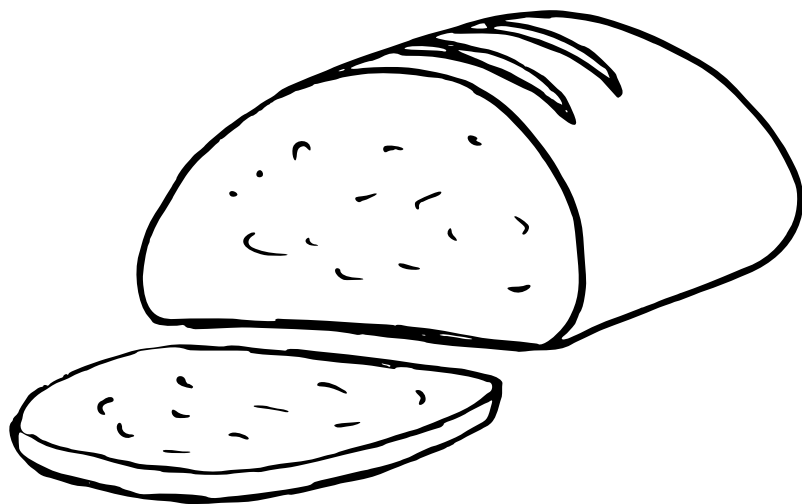

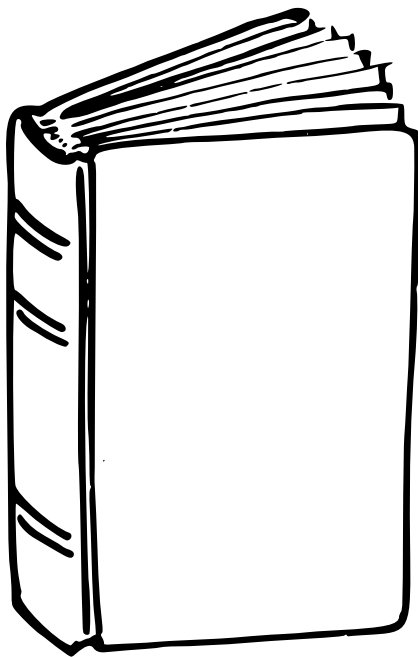

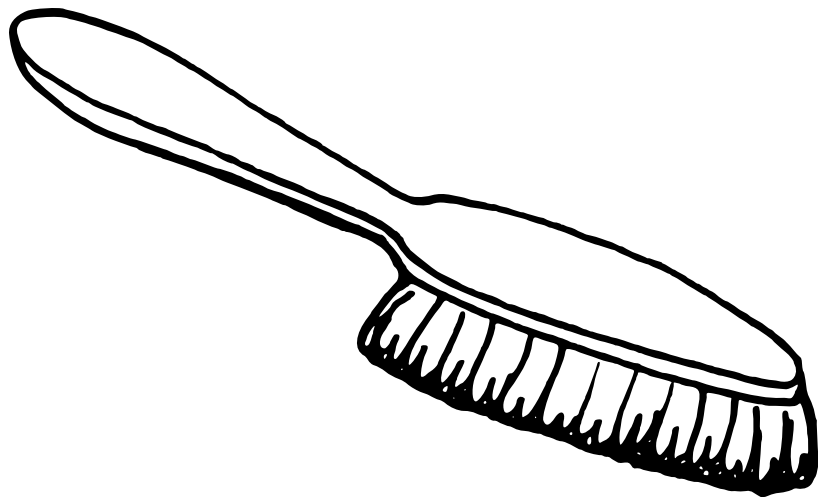

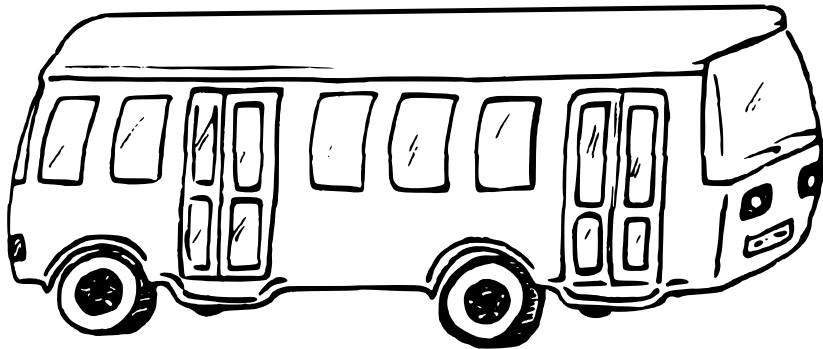

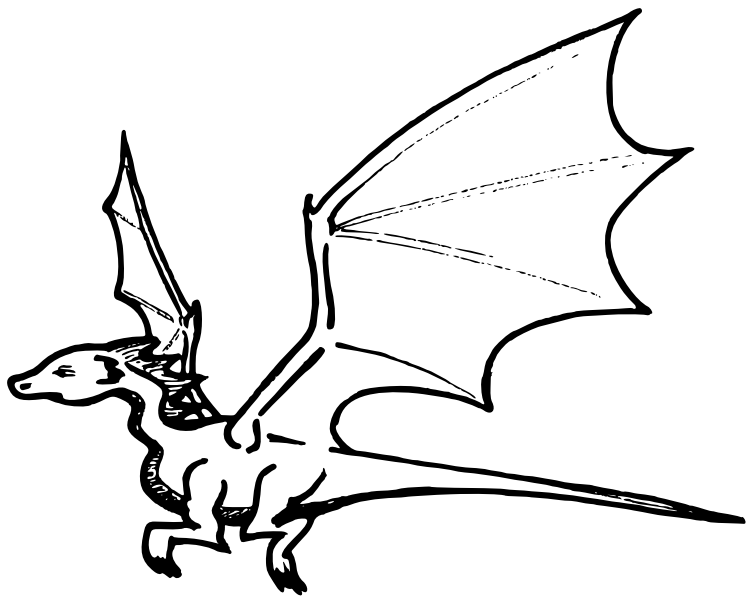

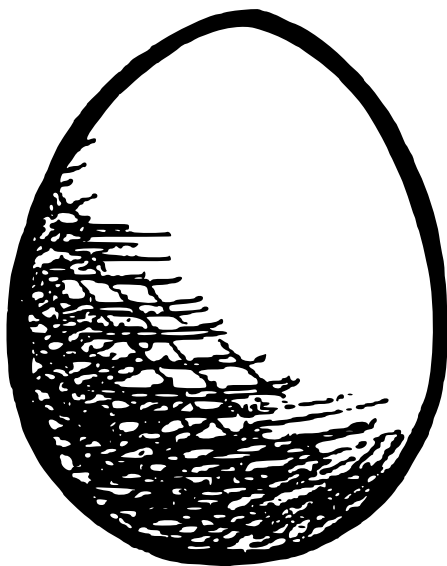

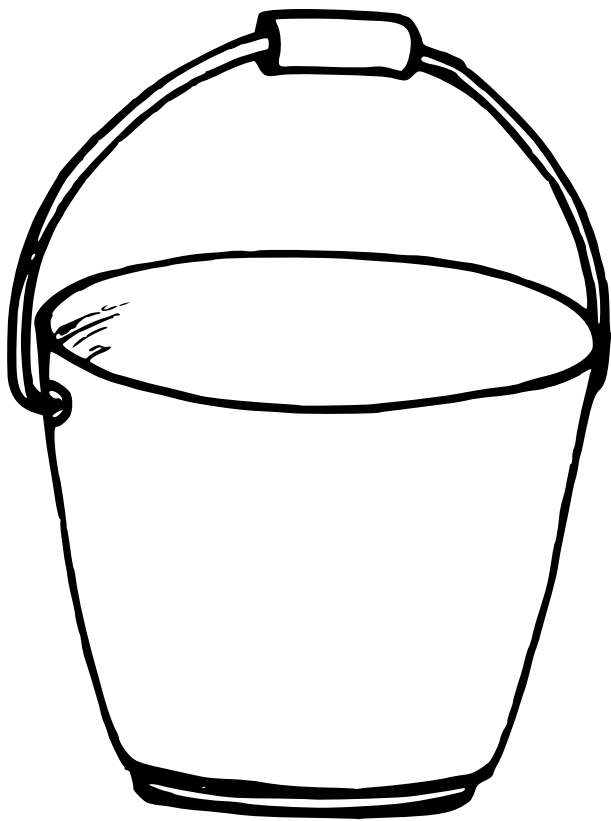

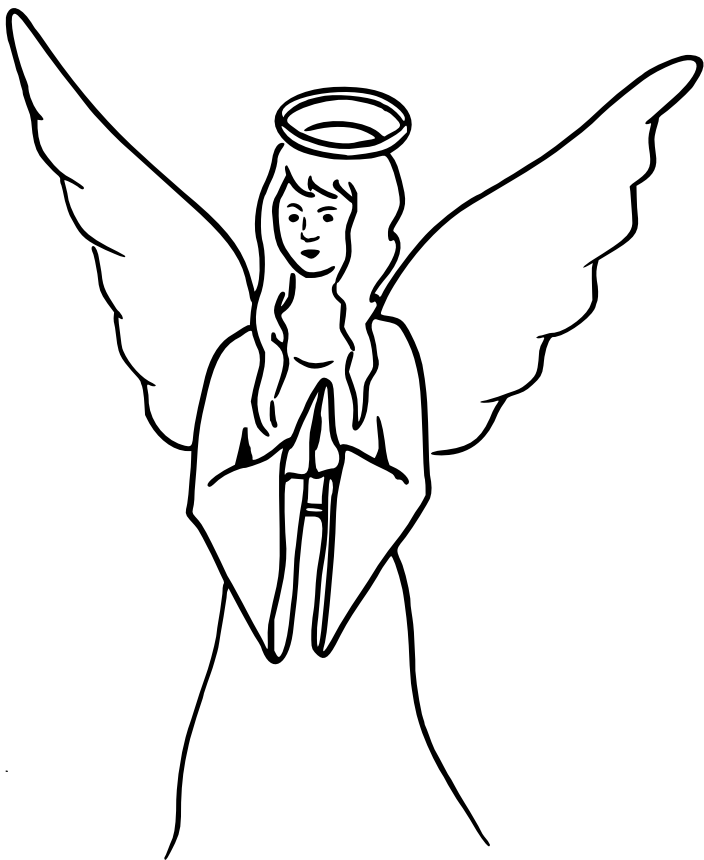

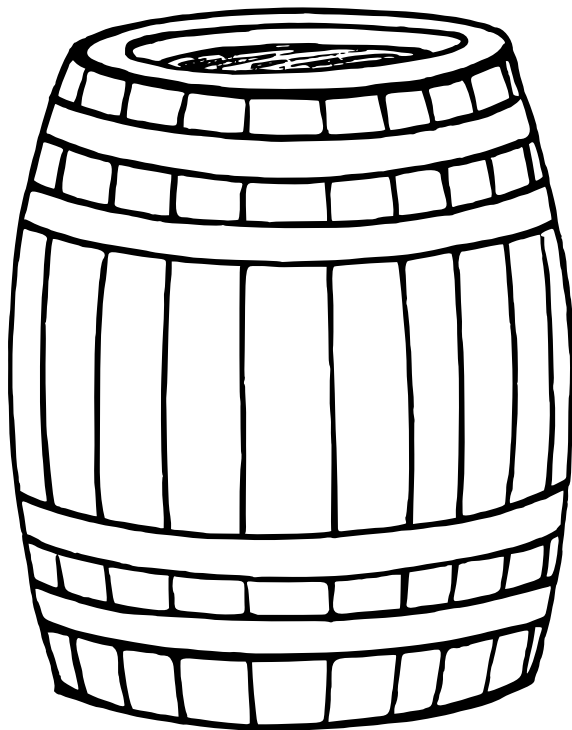

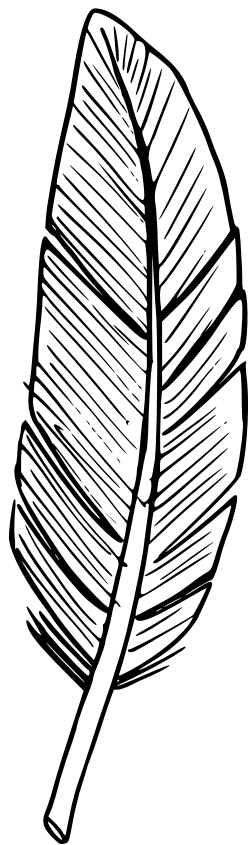

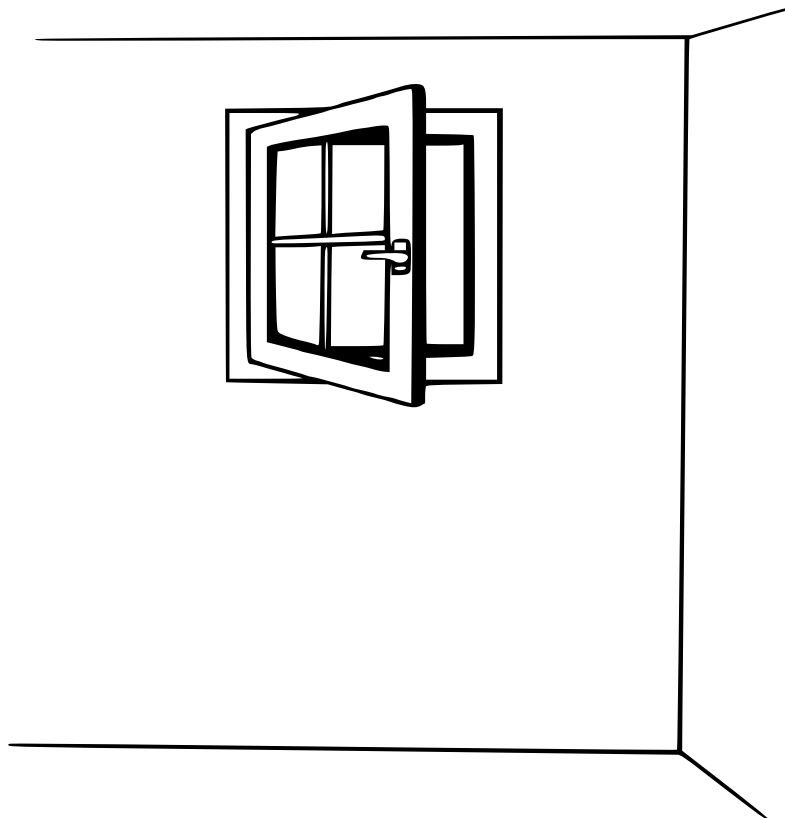

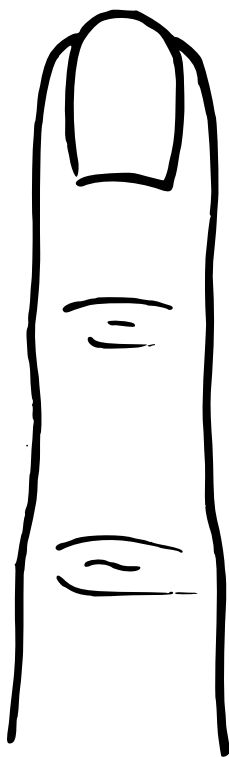

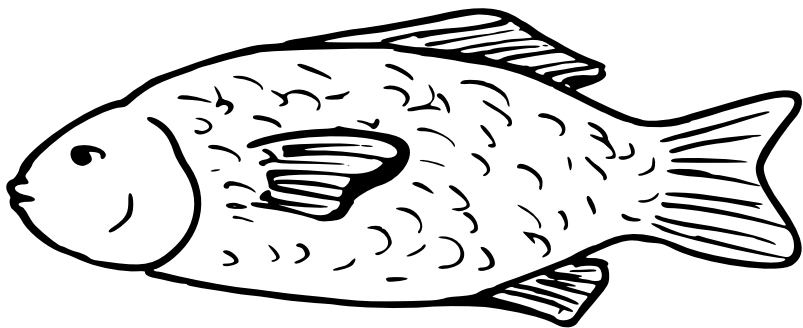

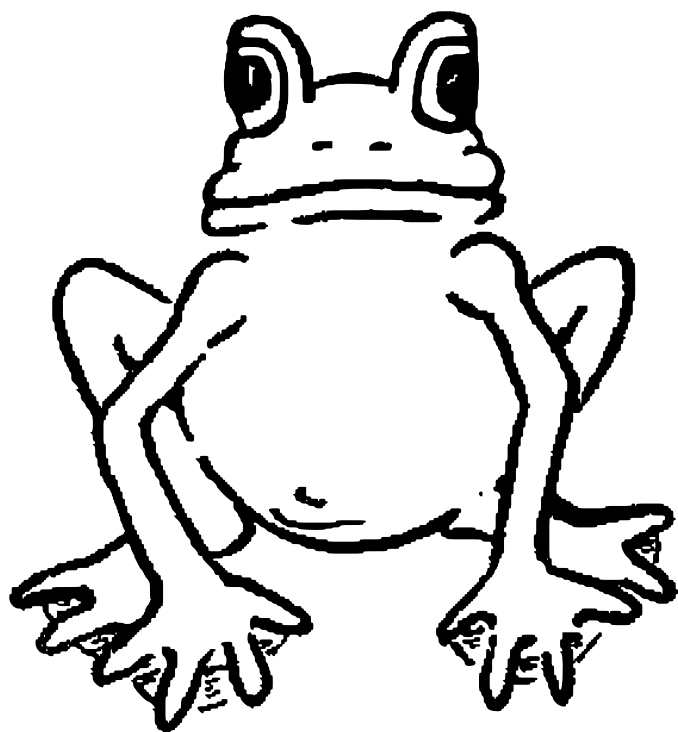

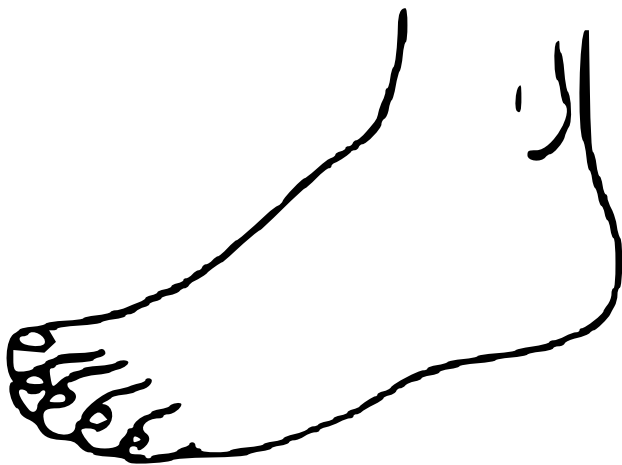

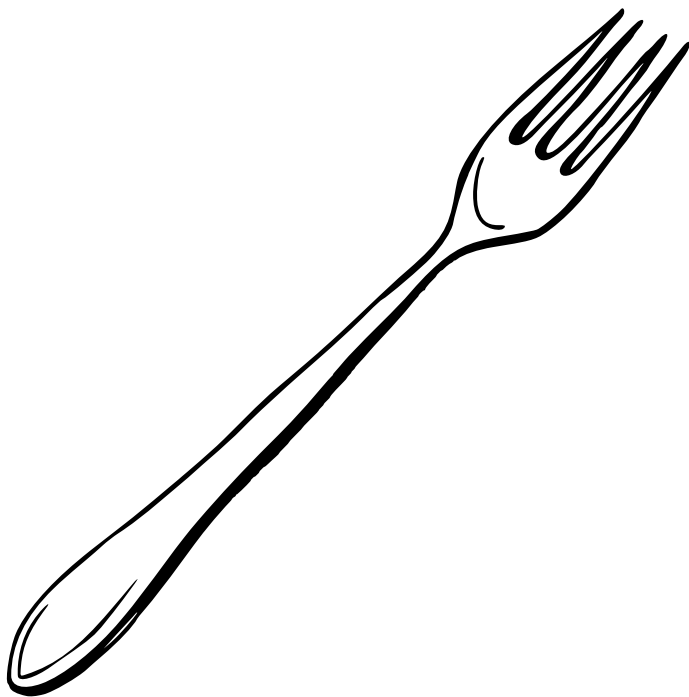

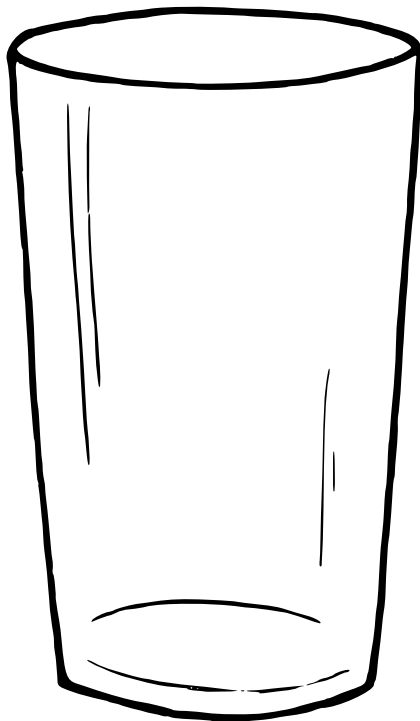

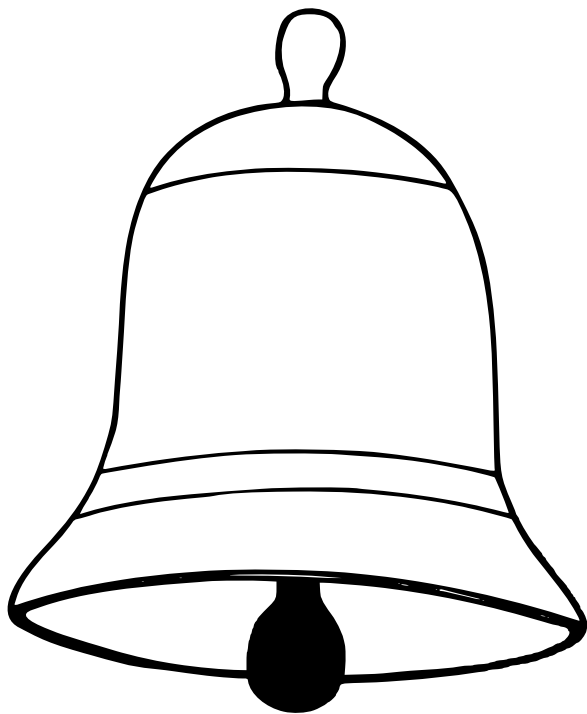

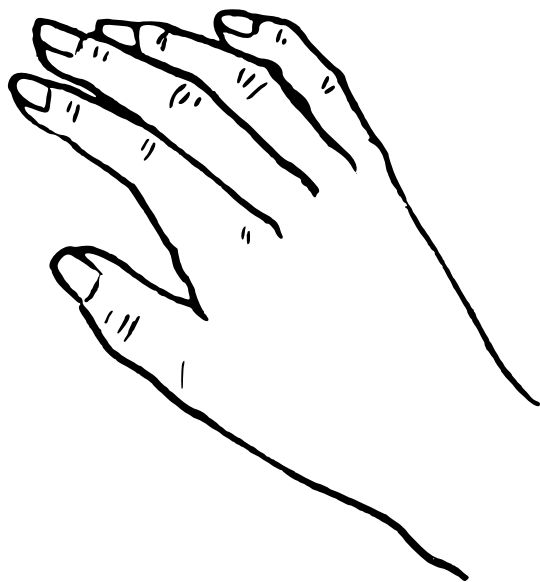

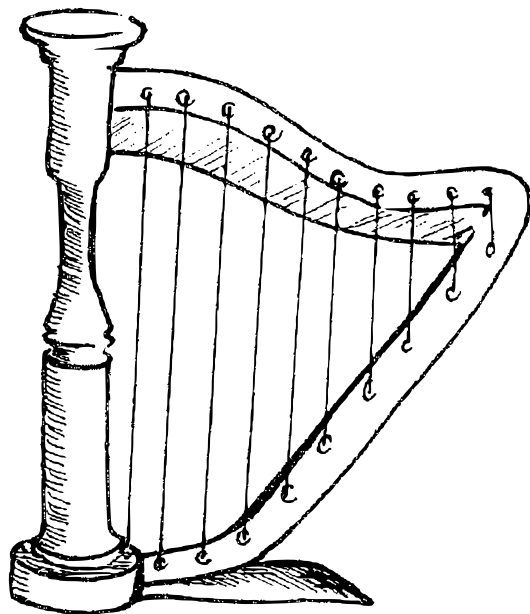

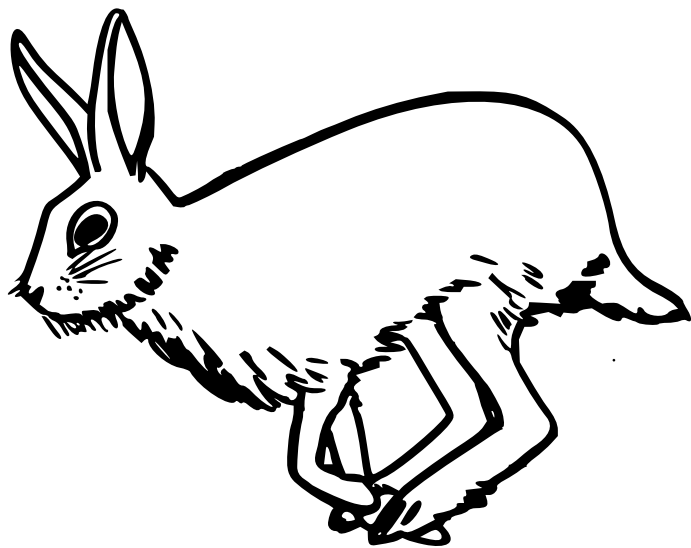

|| u l u u u u u | l l l l ~ ~ ~

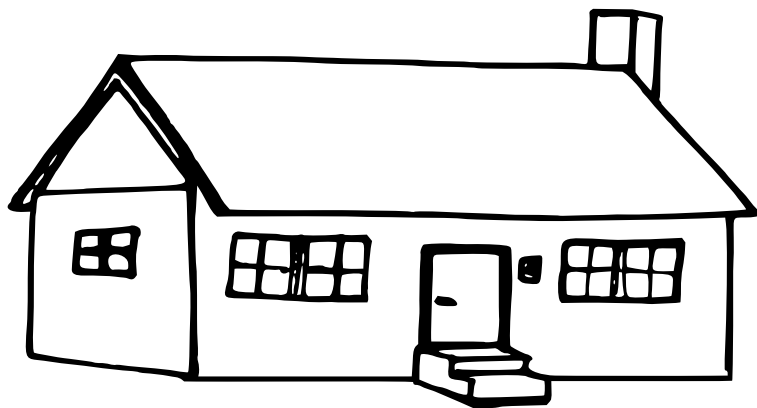

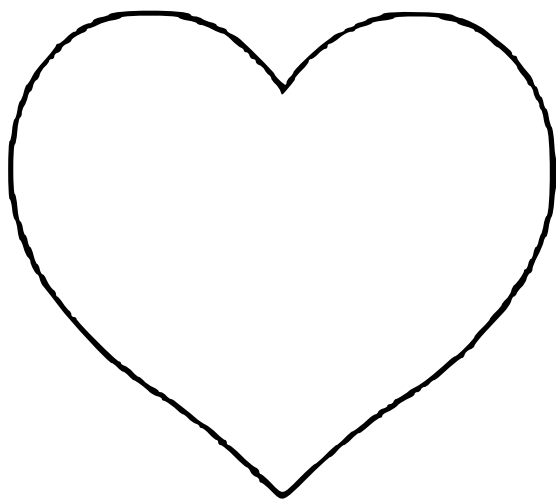

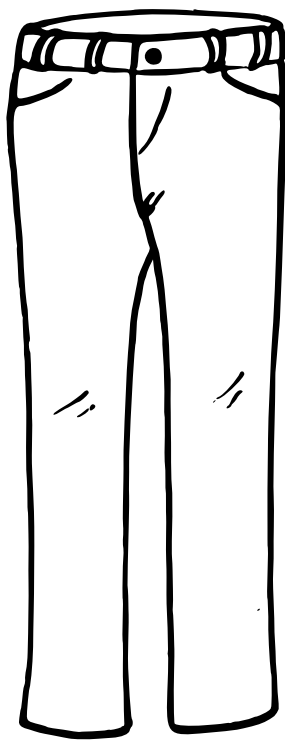

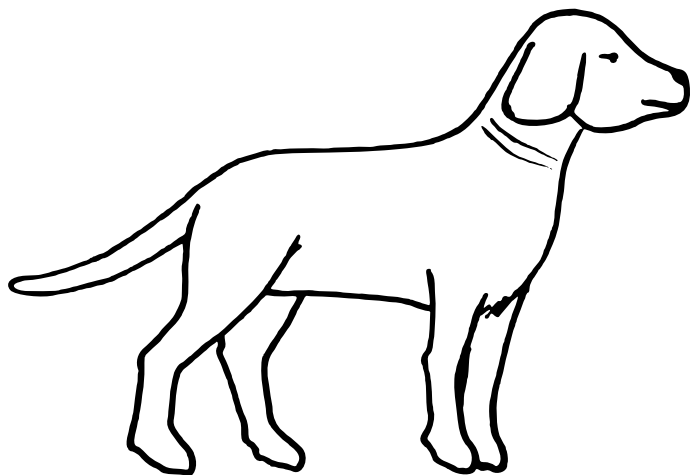

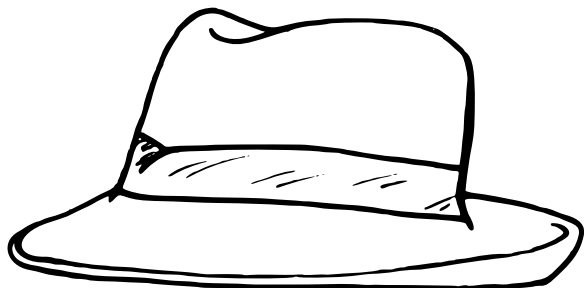

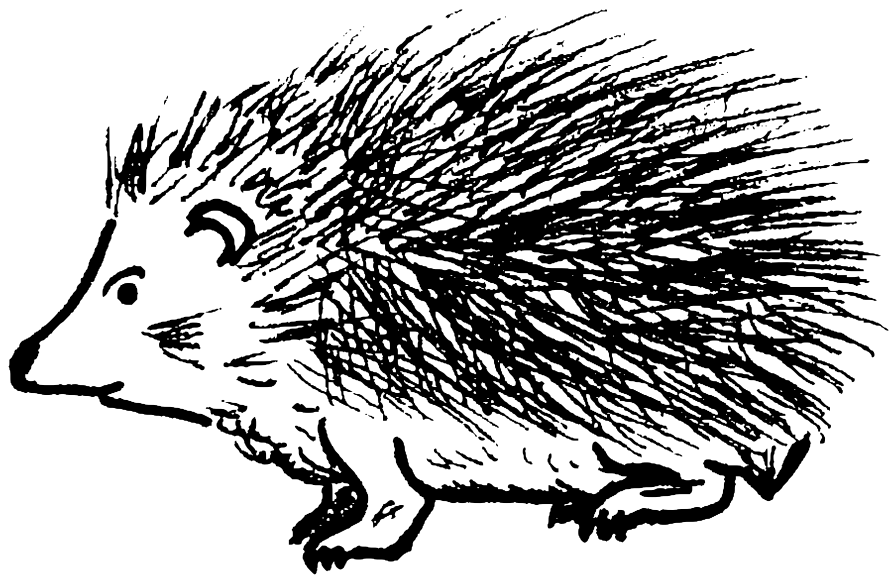

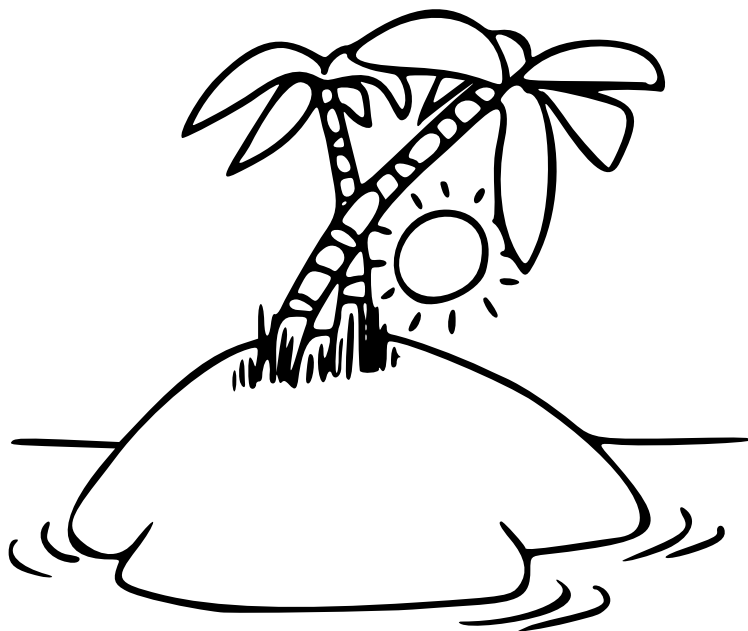

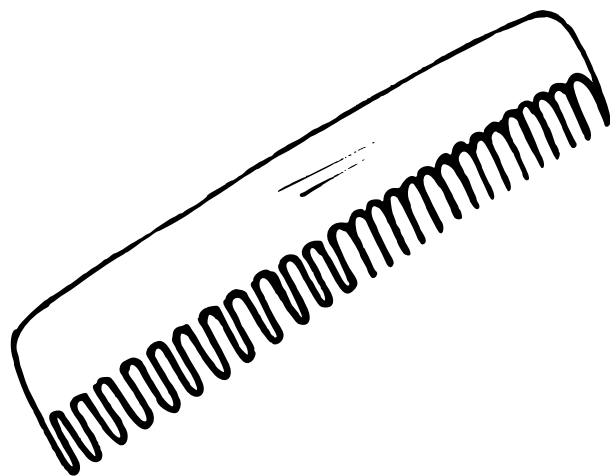

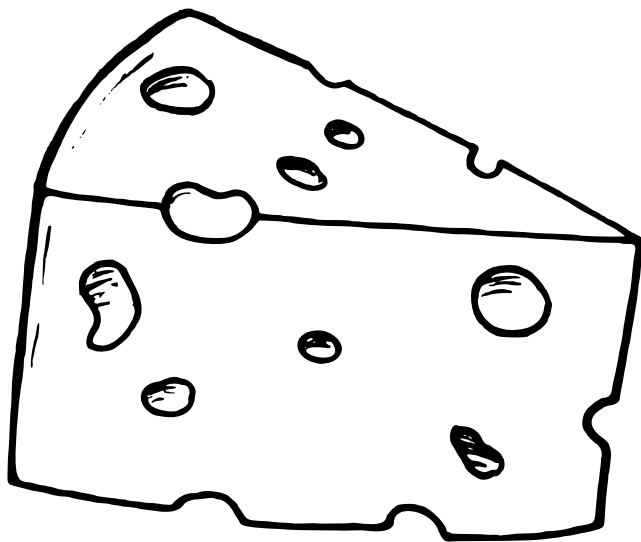

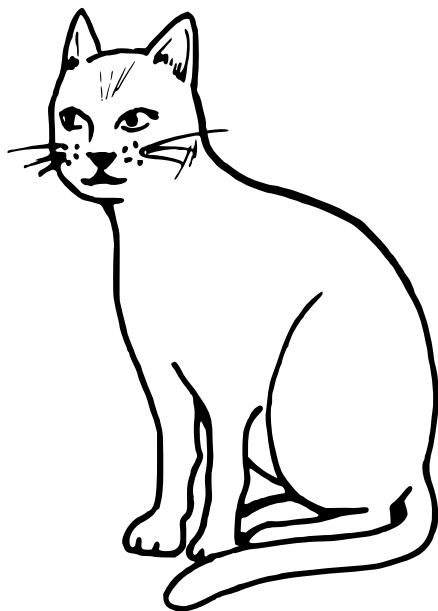

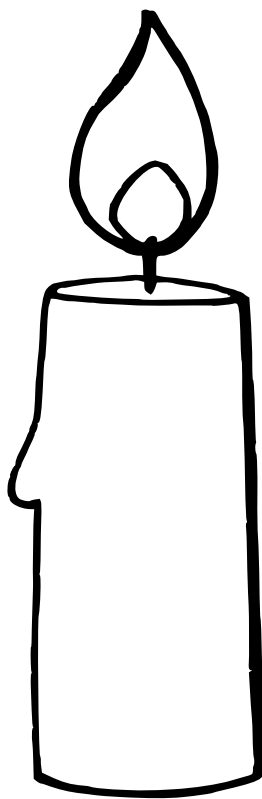

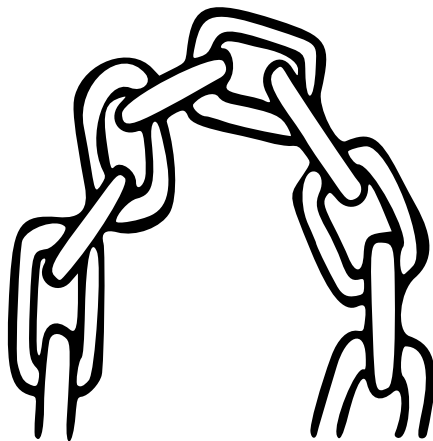

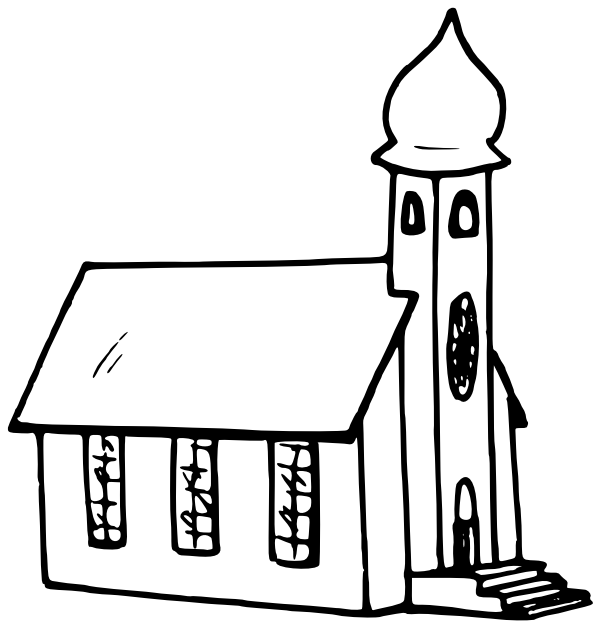

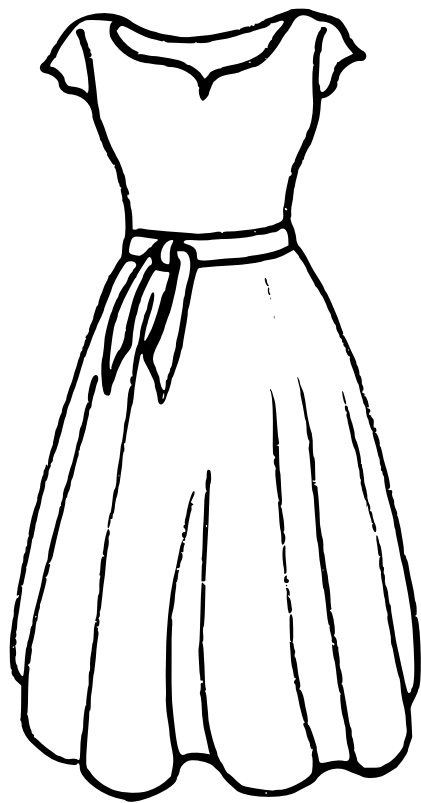

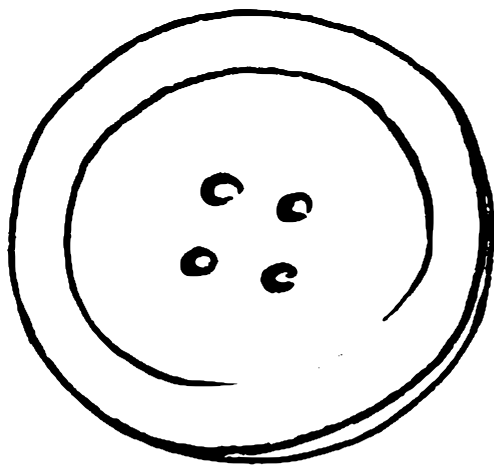

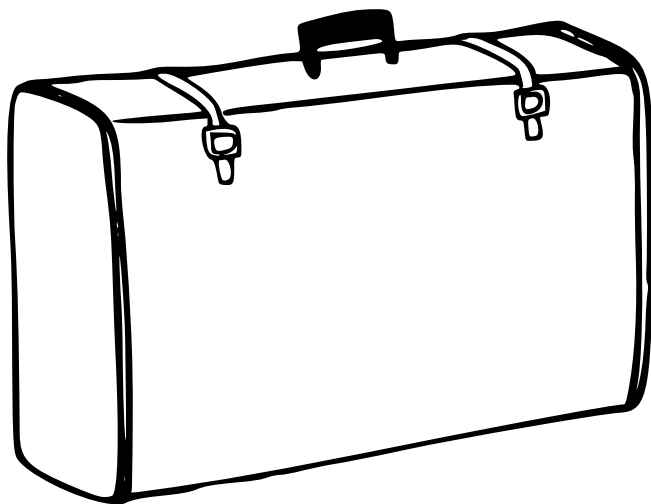

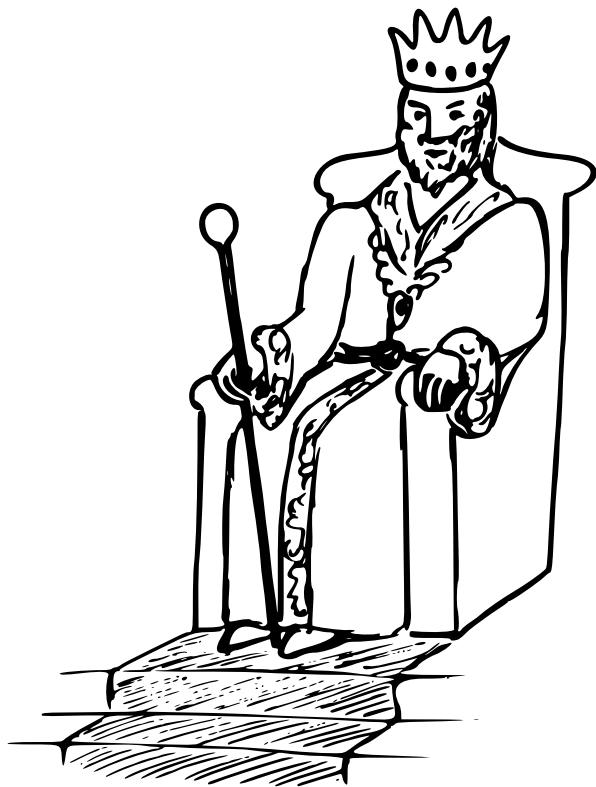

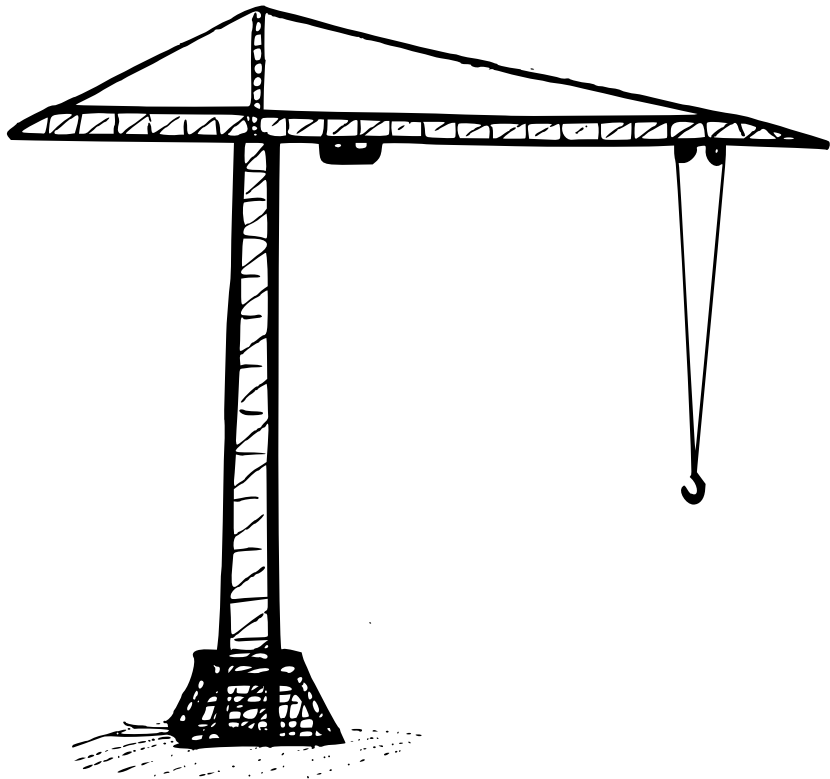

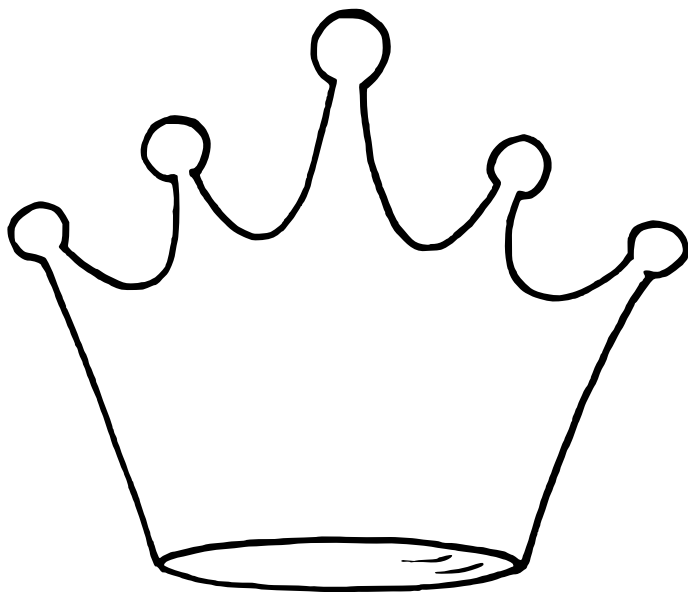

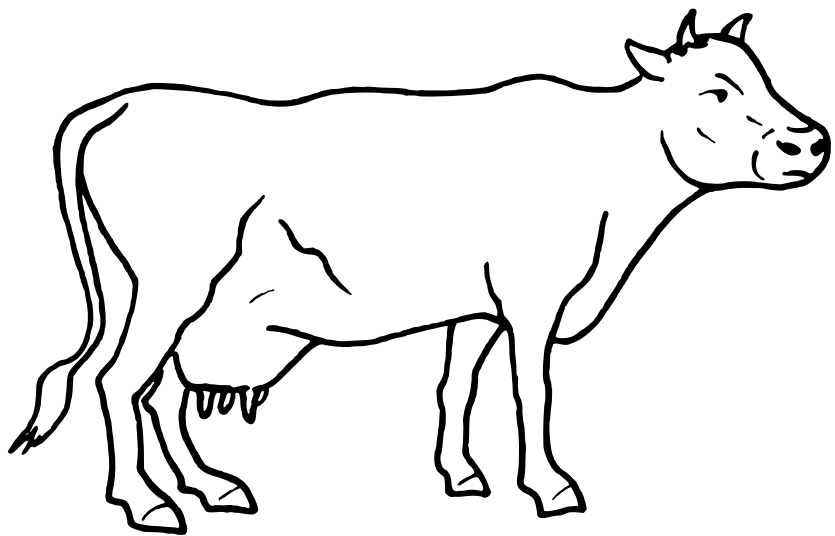

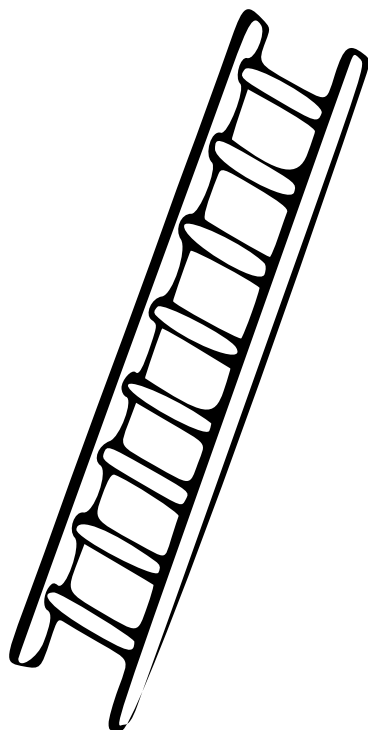

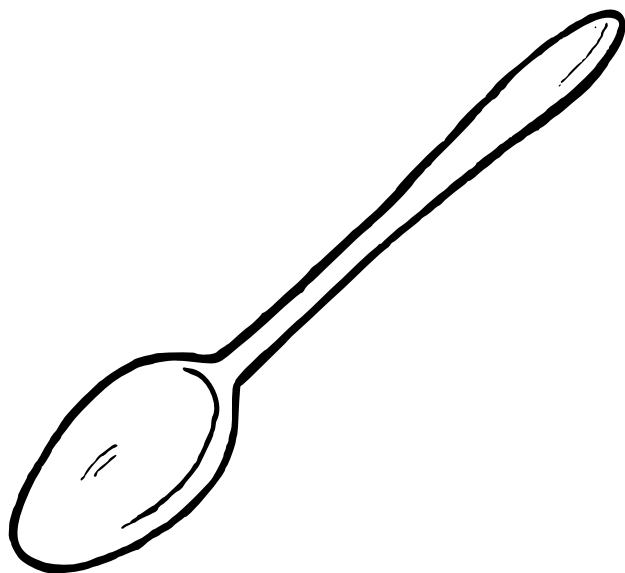

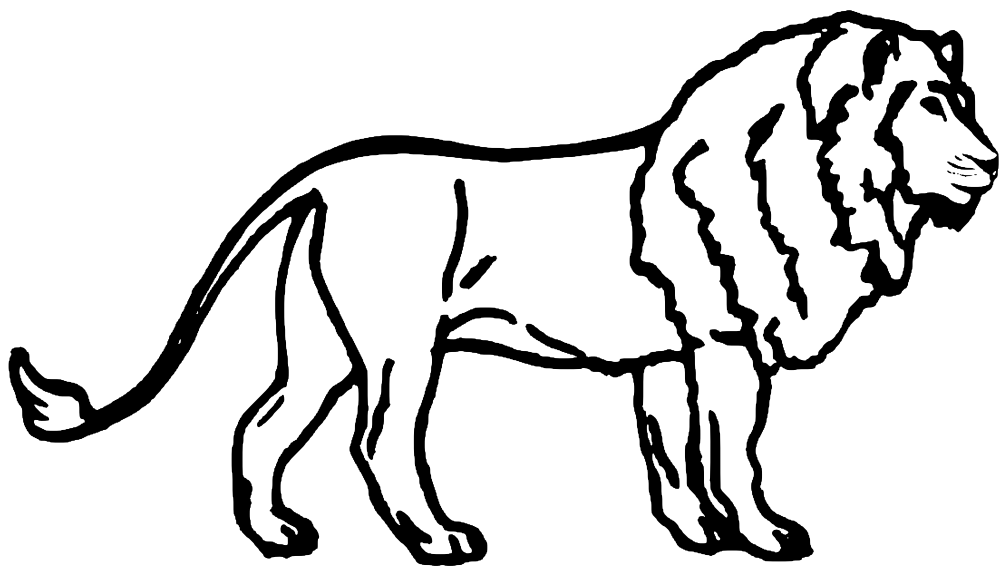

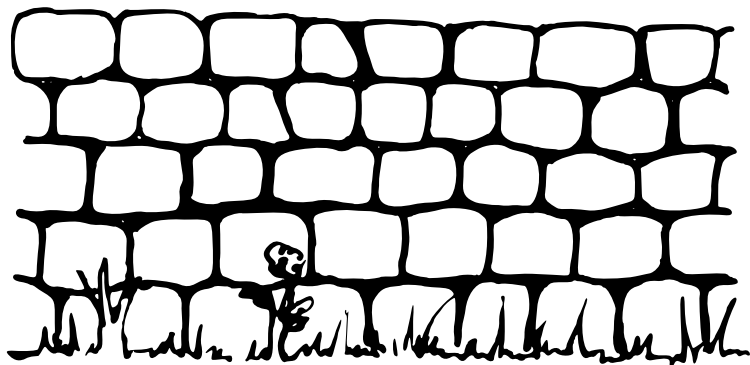

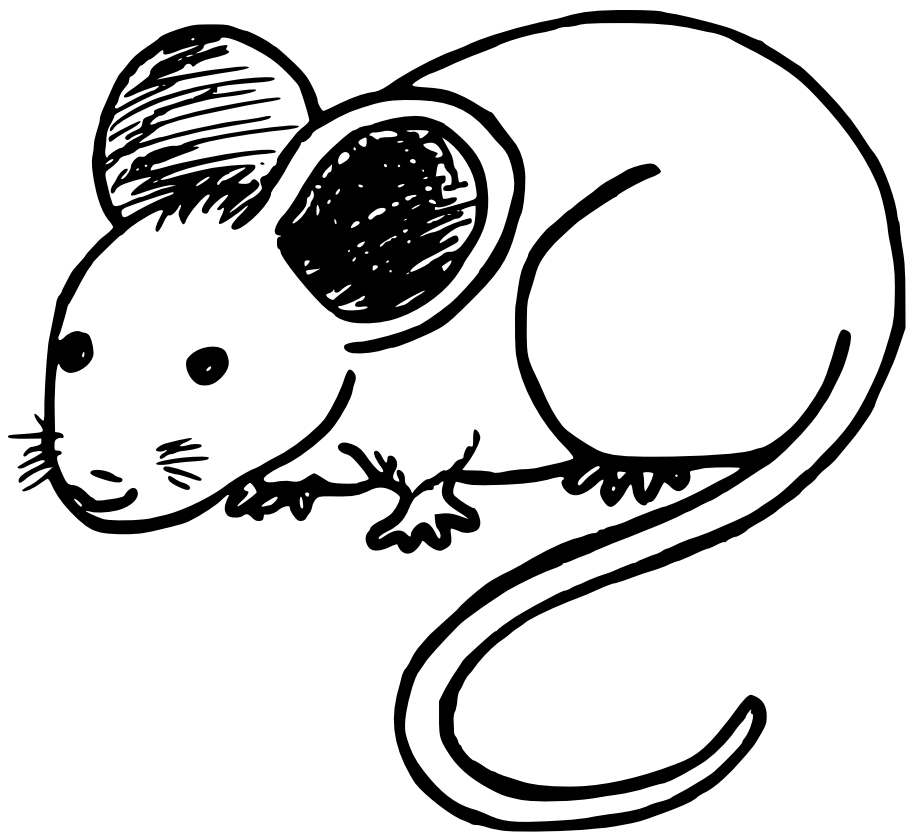

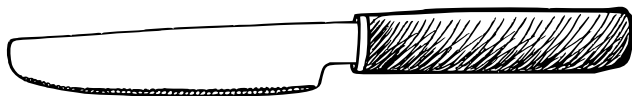

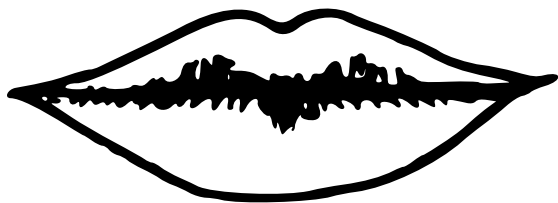

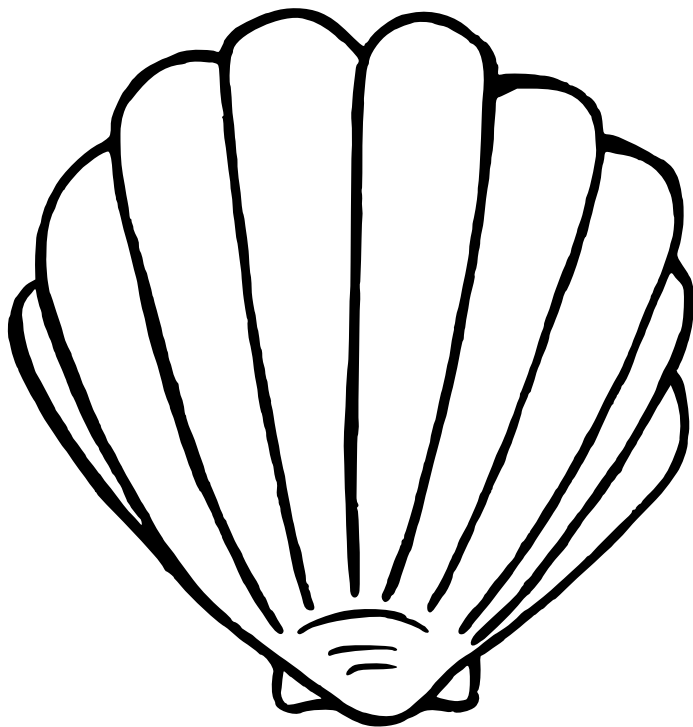

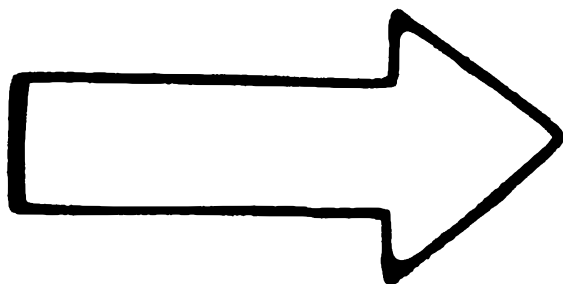

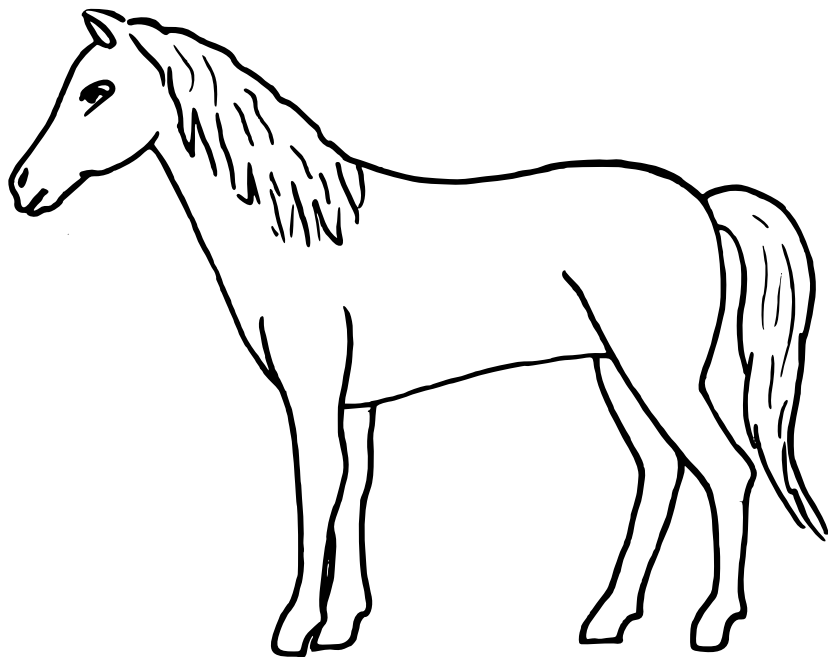

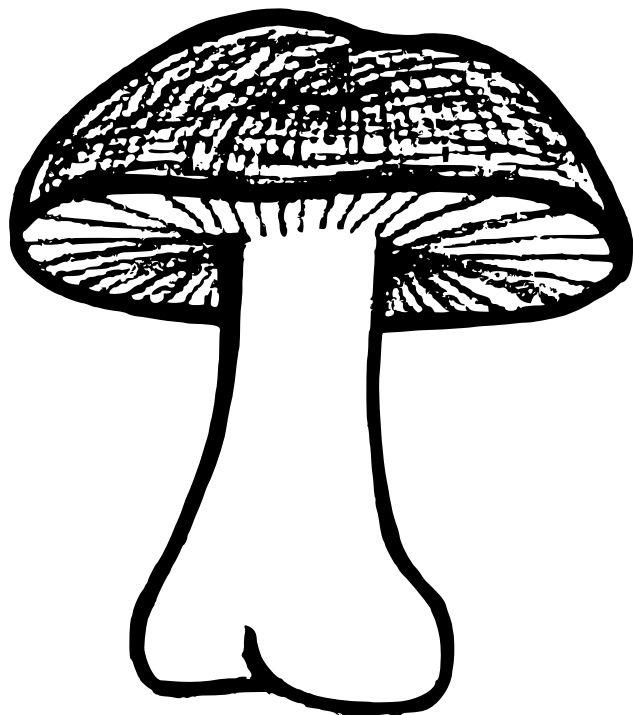

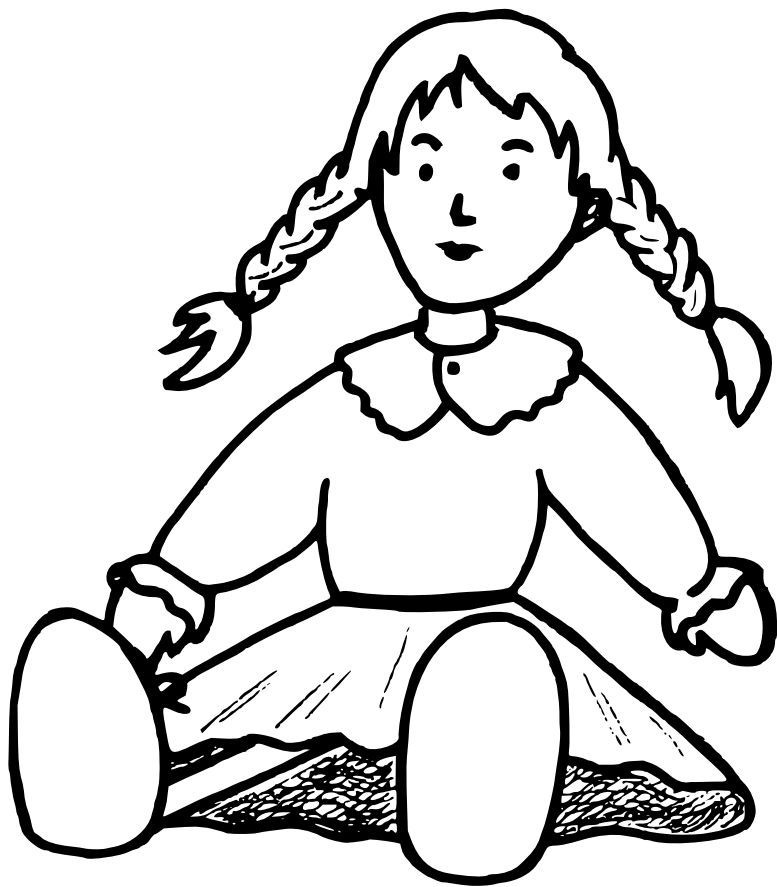

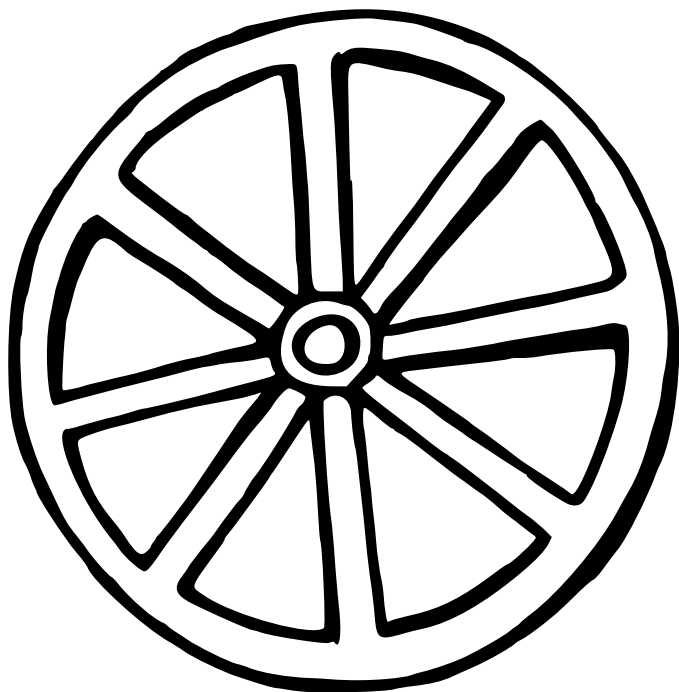

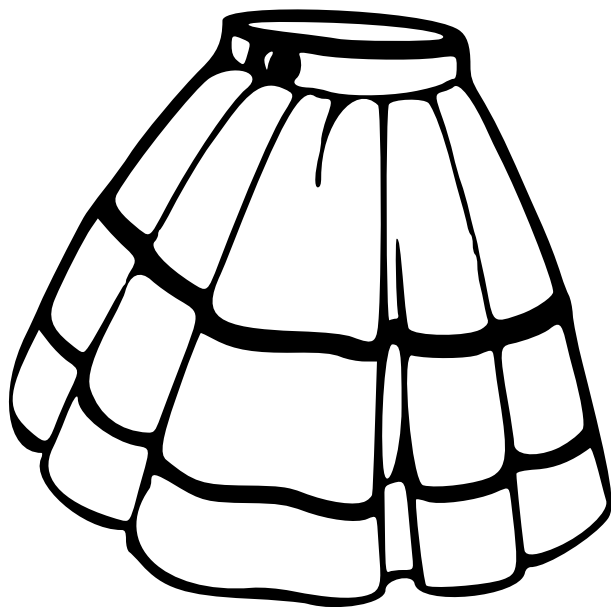

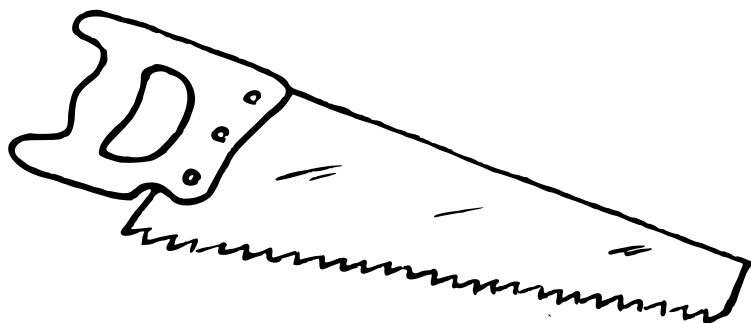

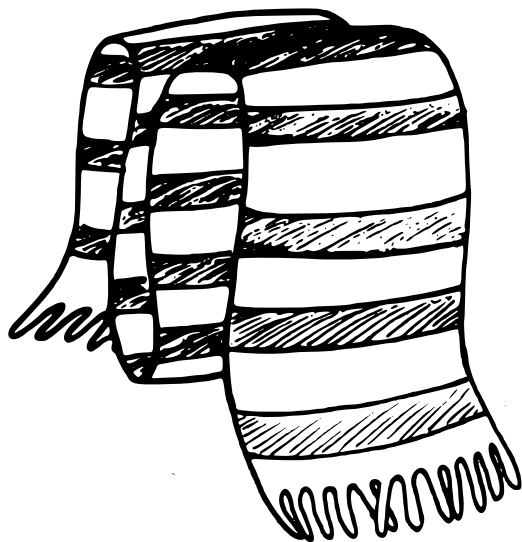

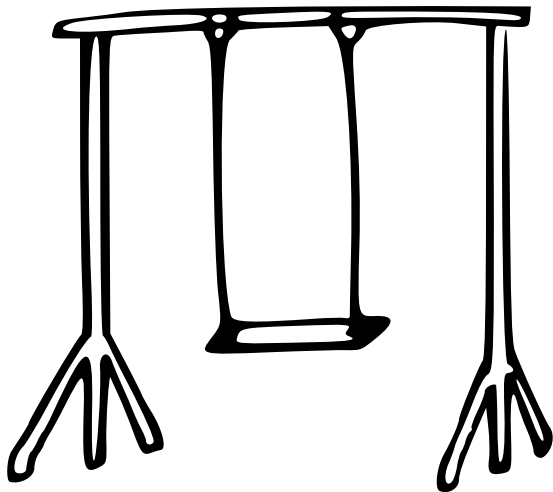

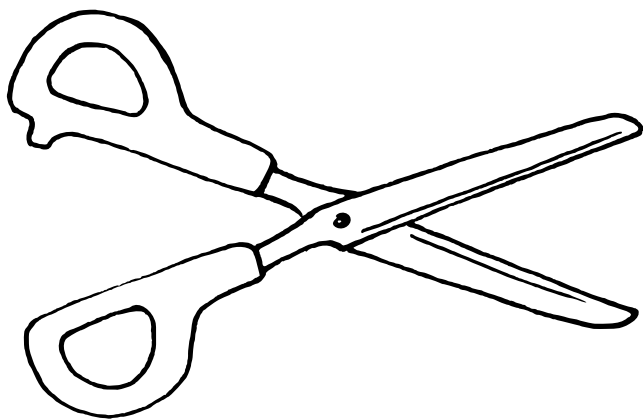

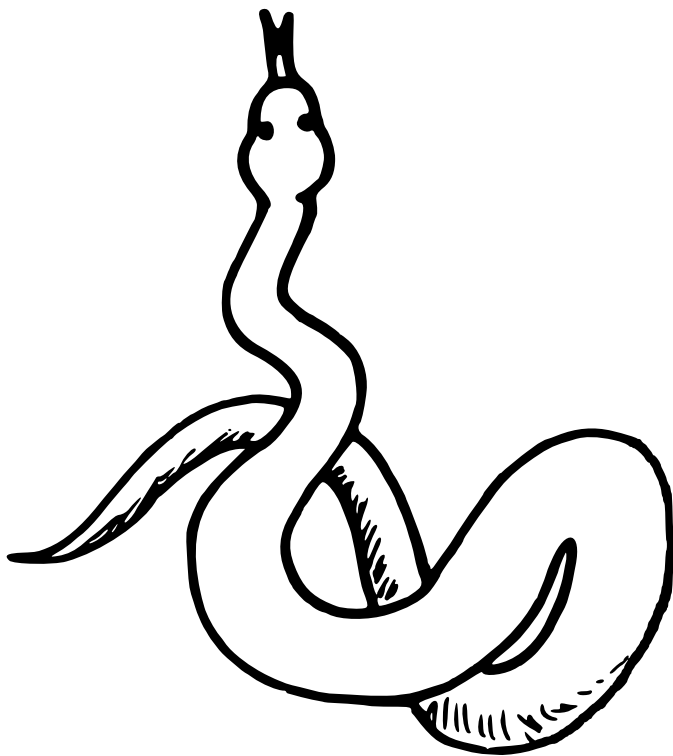

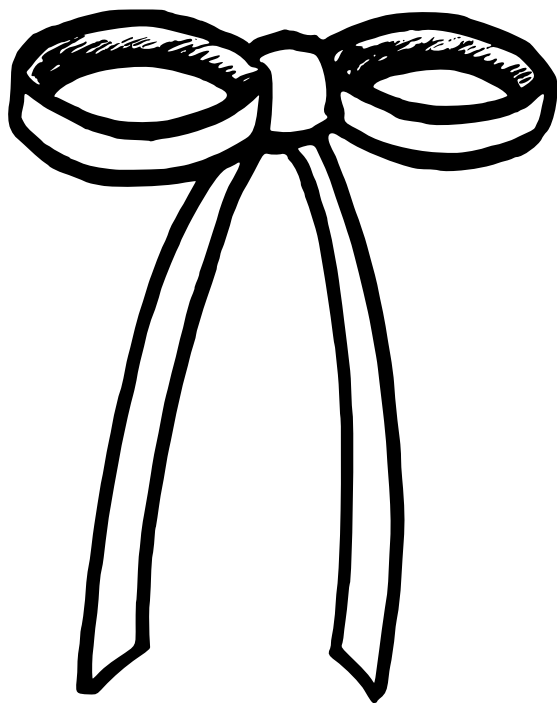

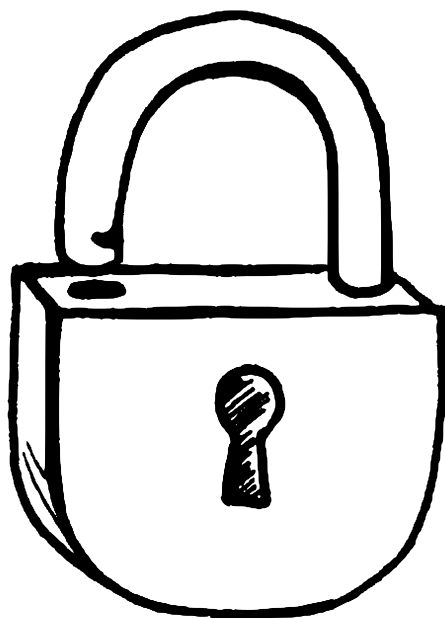

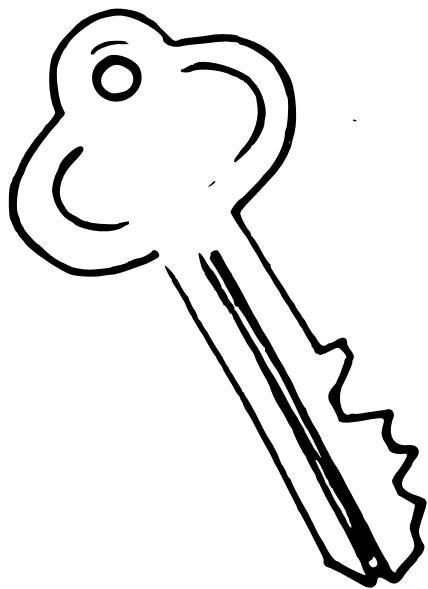

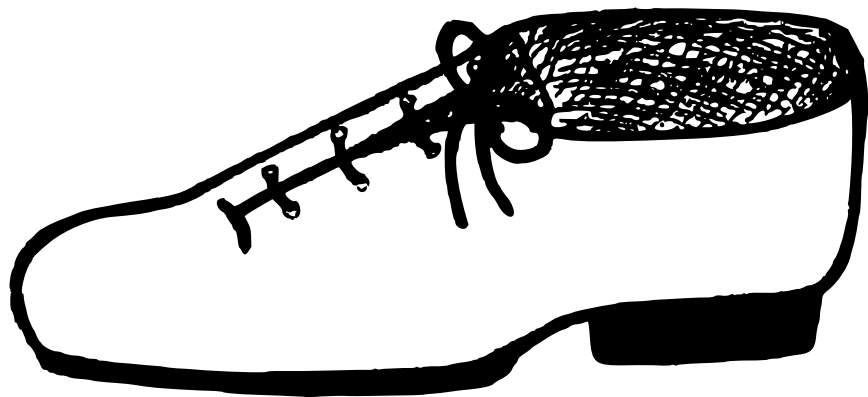

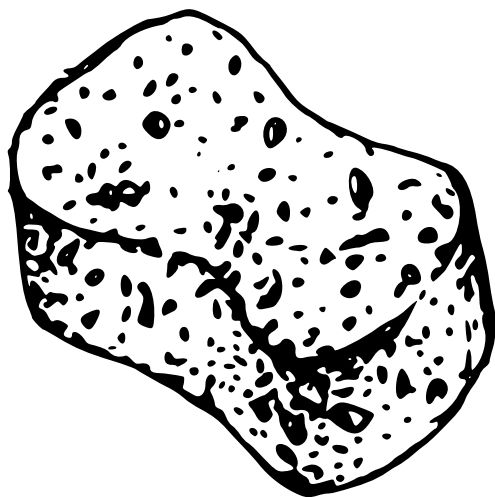

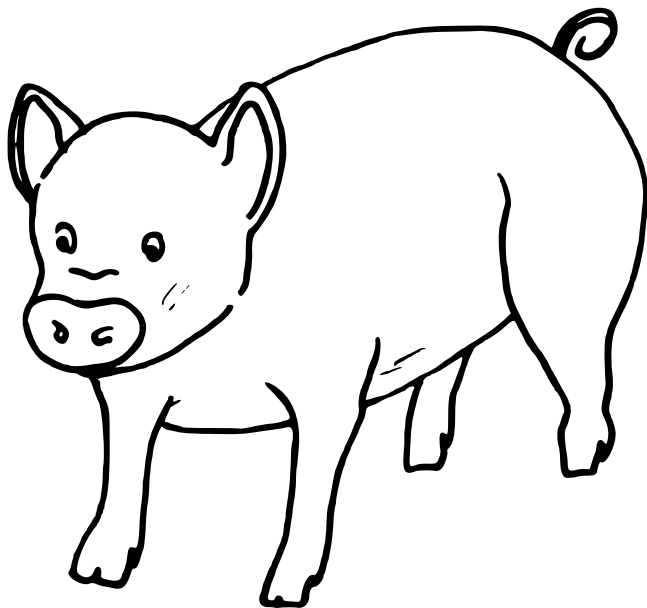

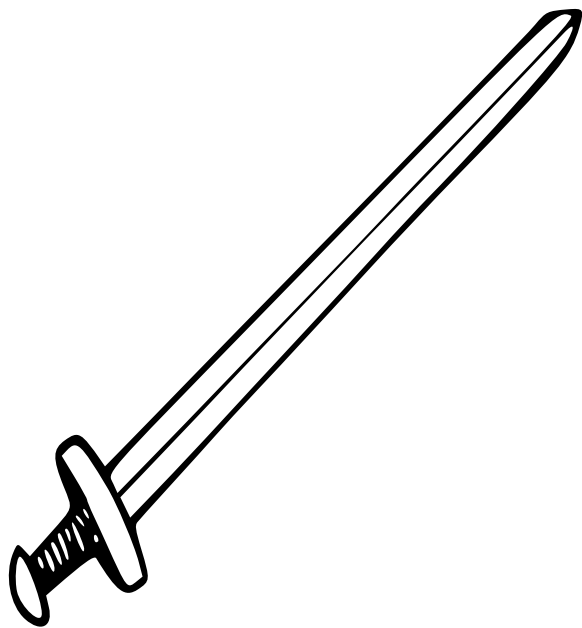

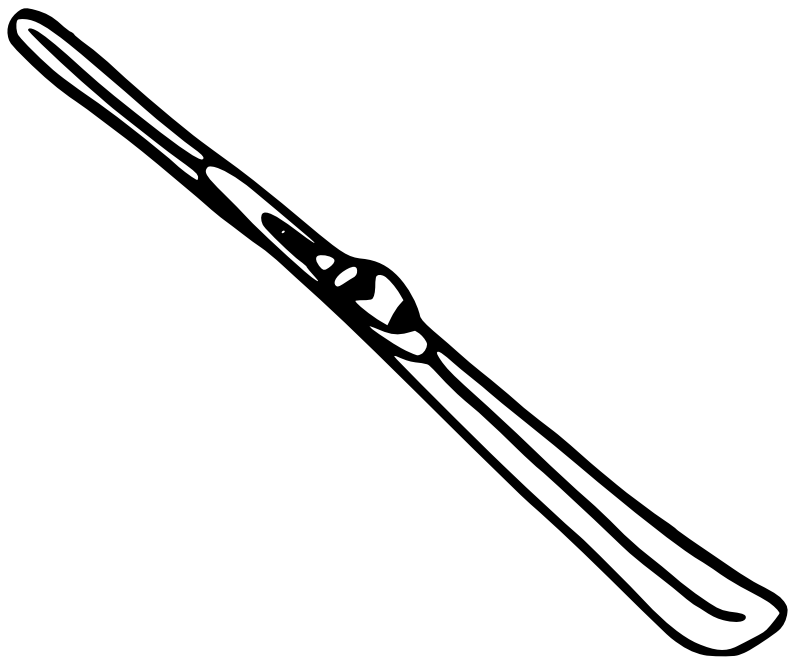

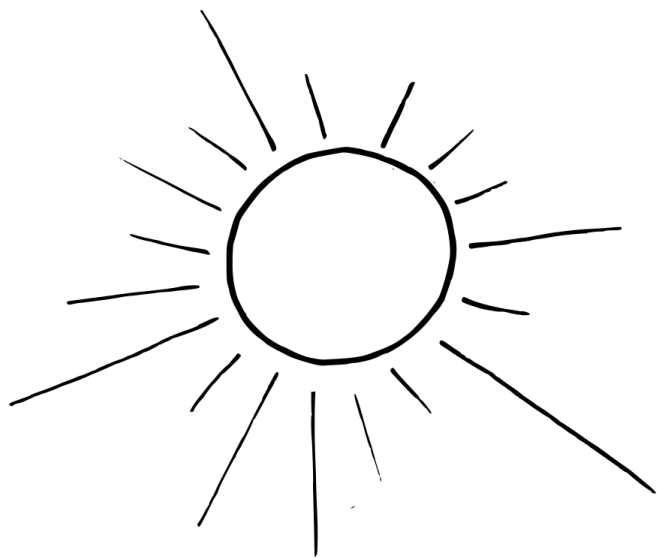

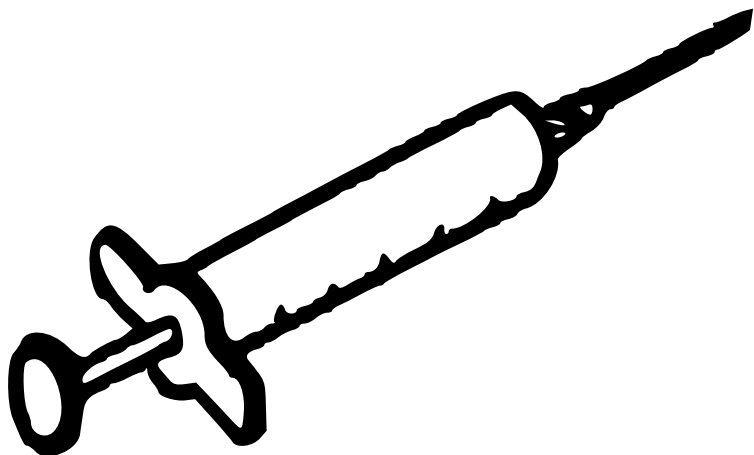

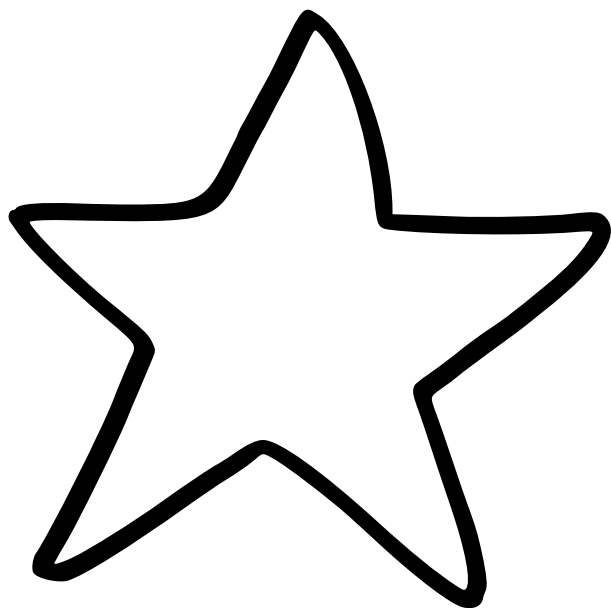

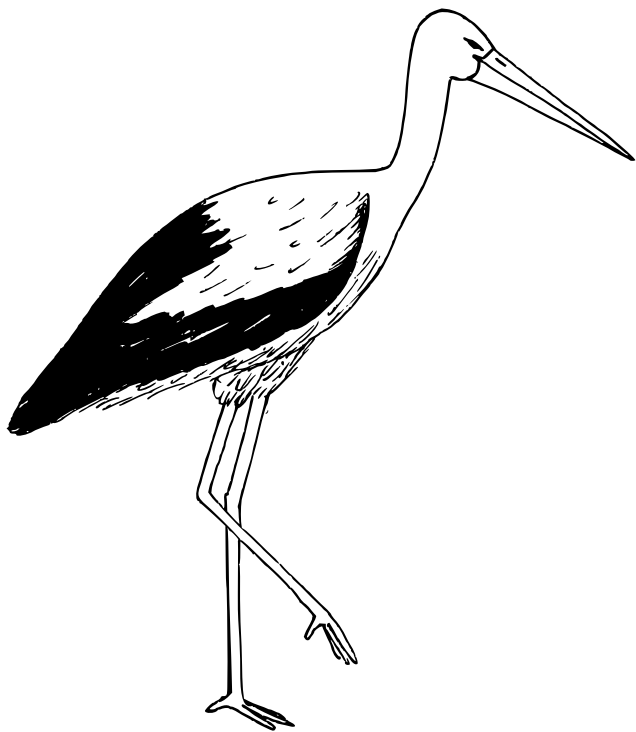

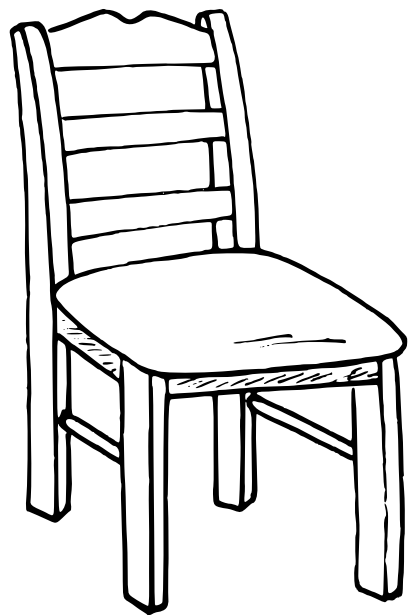

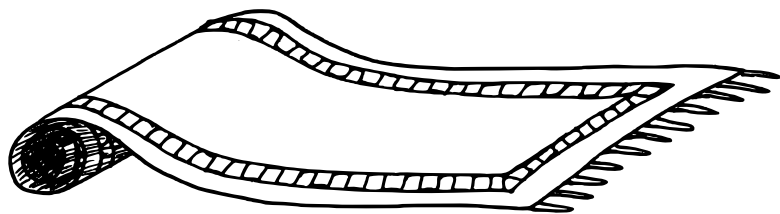

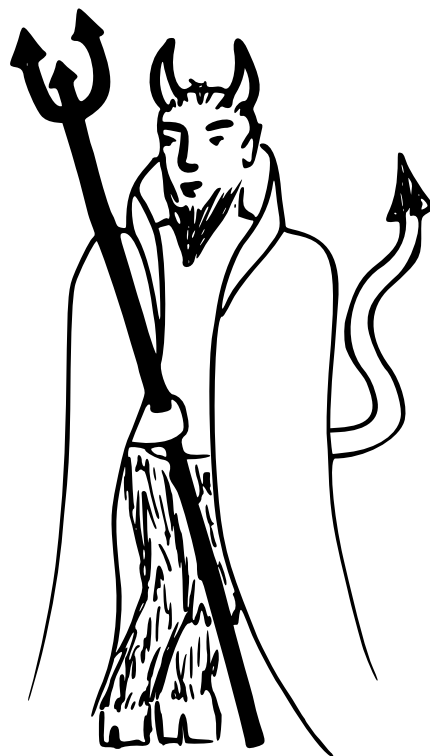

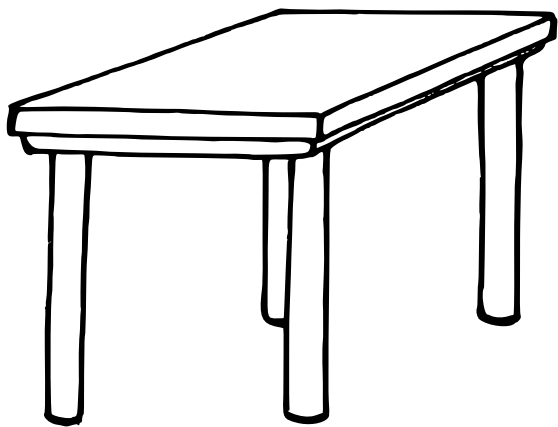

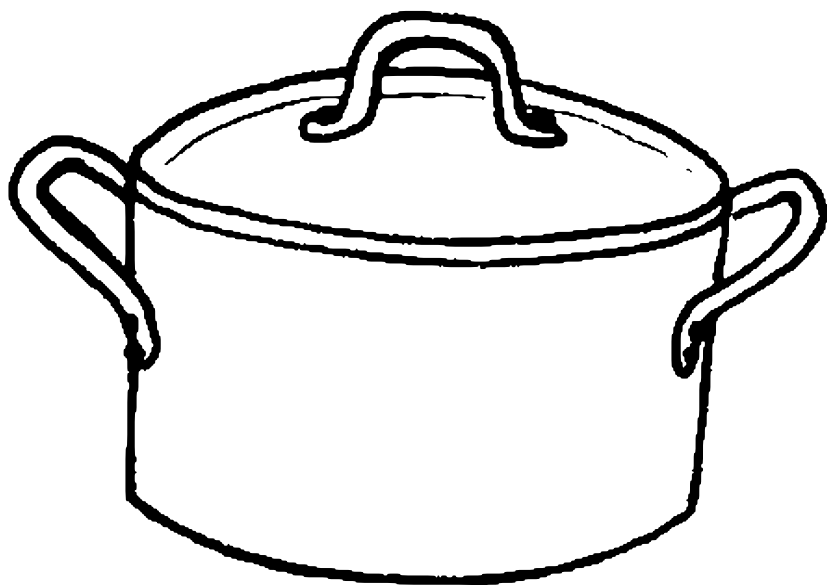

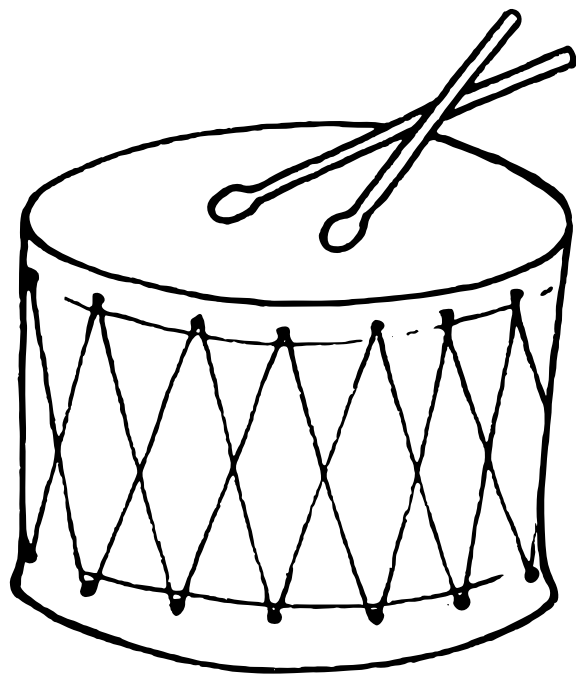

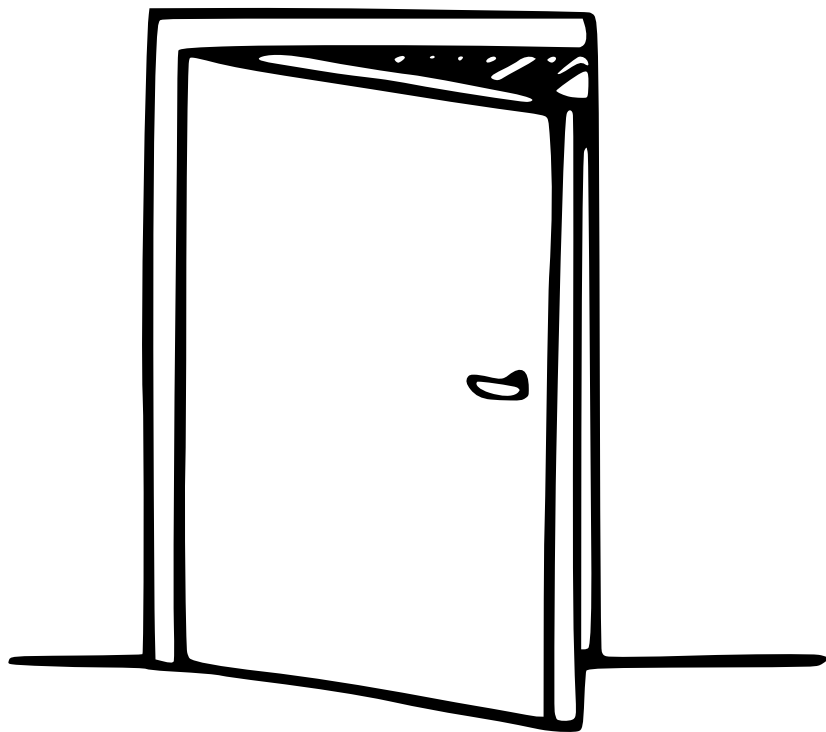

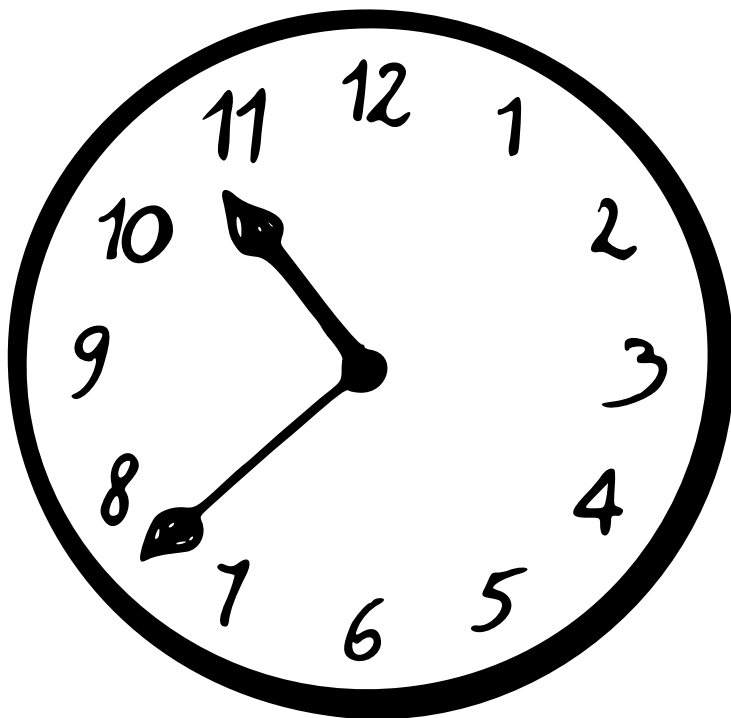

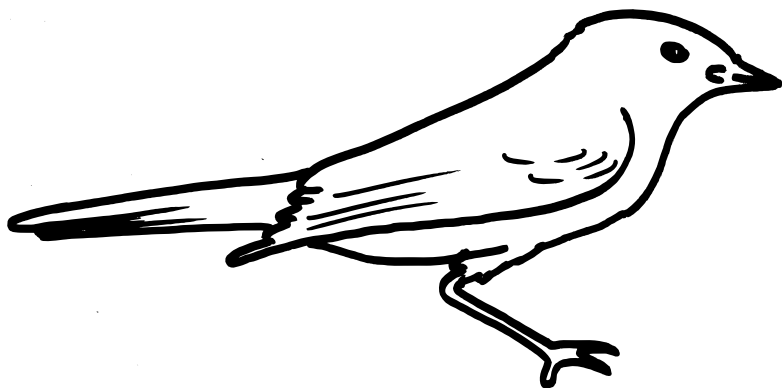

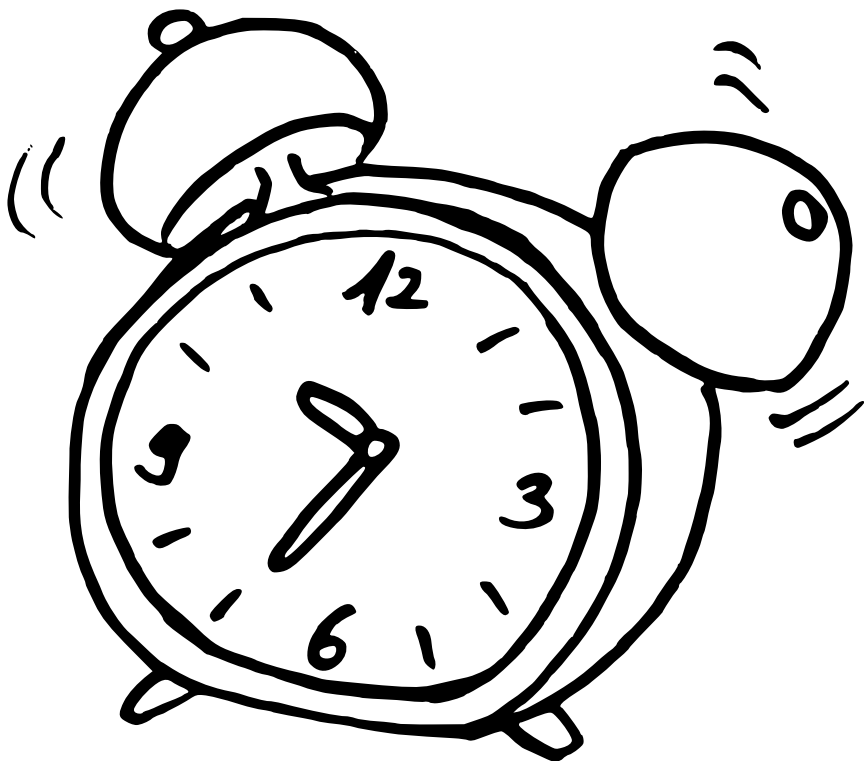

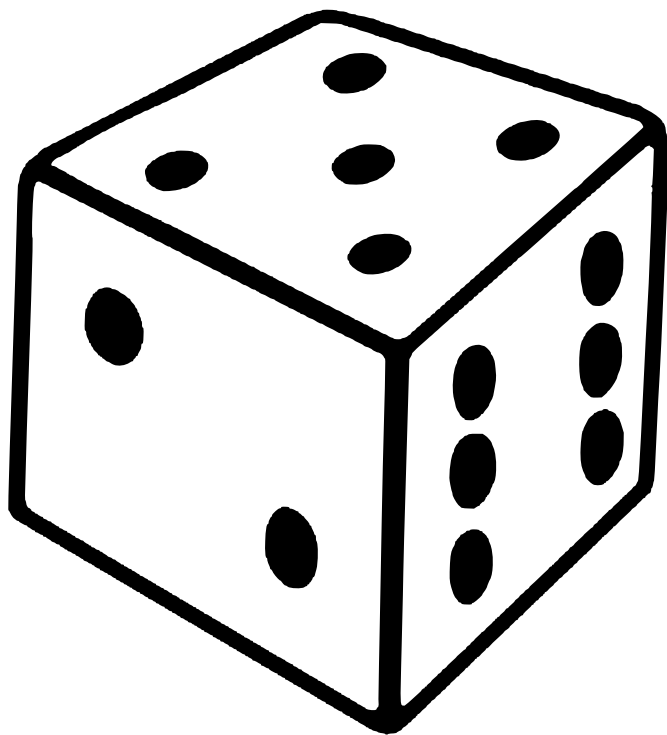

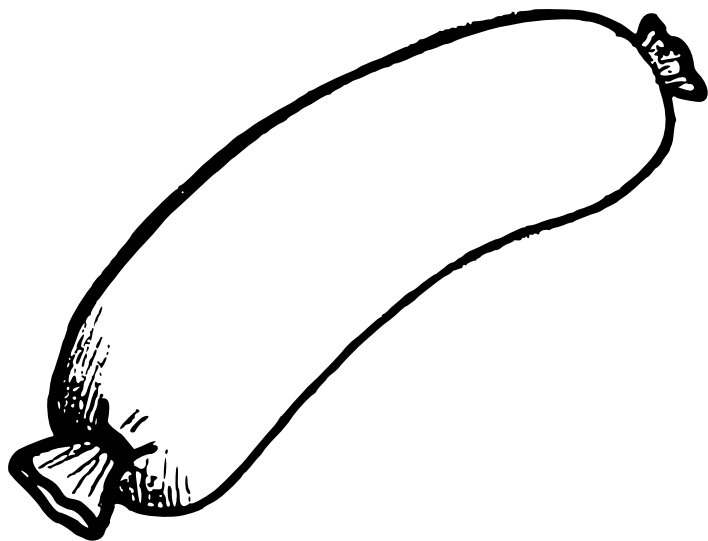

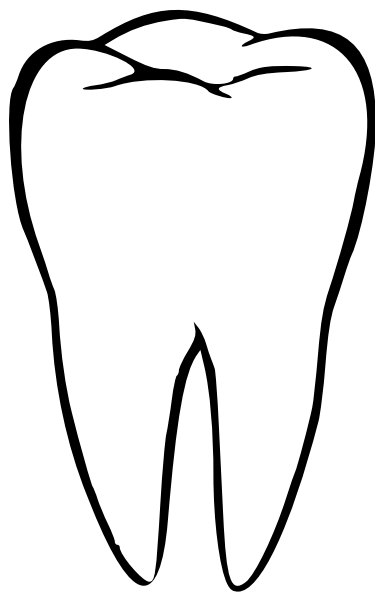

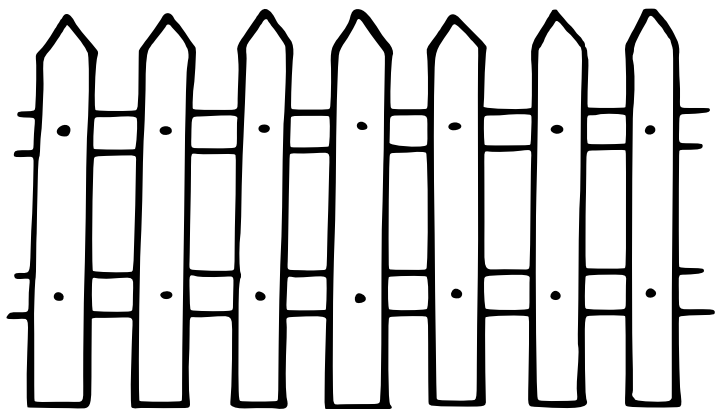

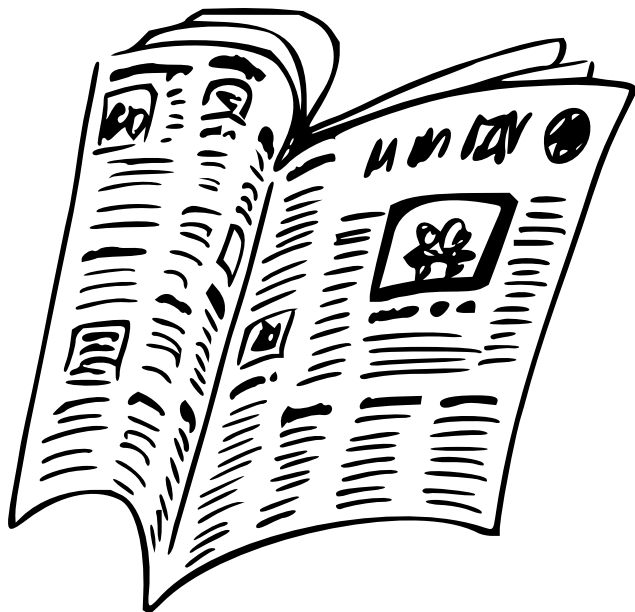

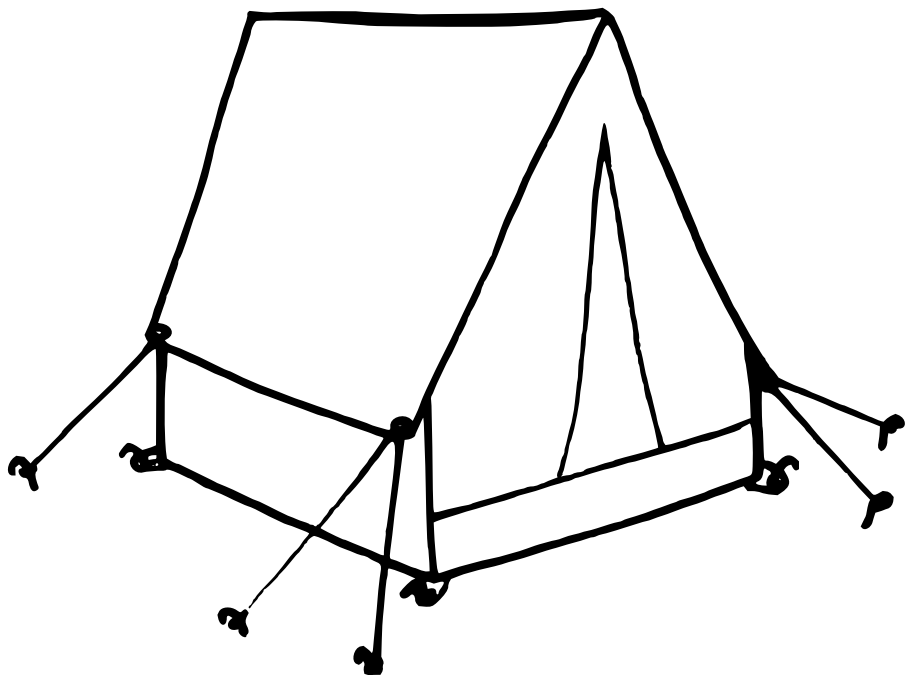

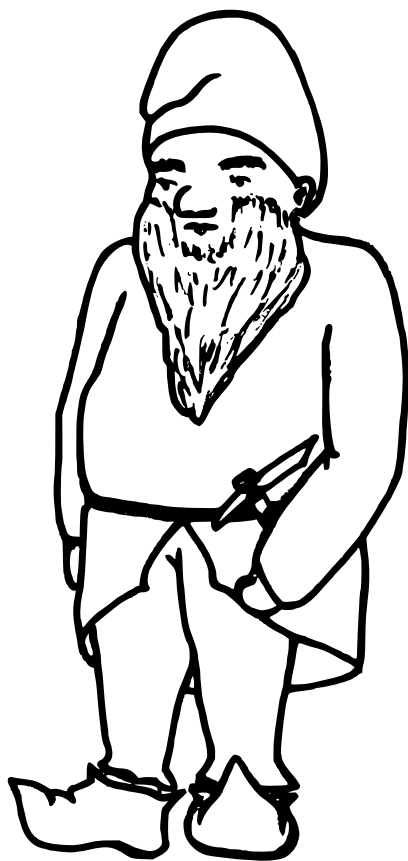

Supplement: Supplementary file 5 [file Data_Sheet_3.pdf]
